# Engineered probiotics for inflammatory bowel disease therapy: mechanisms, delivery strategies, and precision medicine

**DOI:** 10.3389/fmicb.2025.1696524

**Published:** 2026-01-20

**Authors:** Xiaohua Wang, Yindi Cheng, Jiahui Huang, Feixuan Xu, Jian Jiang, Nonthaneth Nalinratana, Litong Jin, Ying Xue

**Affiliations:** 1 Zhejiang Key Laboratory for Restoration of Damaged Coastal Ecosystems, Zhejiang International Science and Technology Cooperation Base for Biomass Resources Development and Utilization, Taizhou Key Laboratory of Biomedicine and Advanced Dosage Forms, School of Life Sciences, Taizhou University, Taizhou, Zhejiang, China; 2 Department of Emergency Medicine, Taizhou Central Hospital (Taizhou University Hospital), Taizhou, Zhejiang, China; 3 Department of Pharmacology and Physiology, Faculty of Pharmaceutical Sciences, Center of Excellence in Natural Products for Ageing and Chronic Diseases, Chulalongkorn University, Bangkok, Thailand

**Keywords:** inflammatory bowel disease, engineered probiotics, ulcerative colitis, Crohn’s disease, genetic engineering, precision medicine, gut microbiome, biosafety

## Abstract

Inflammatory bowel disease (IBD), encompassing ulcerative colitis (UC) and Crohn’s disease (CD), is a prevalent chronic gastrointestinal disorder. Conventional therapies are often limited by adverse effects and suboptimal long-term efficacy. Probiotics have emerged as promising therapeutic alternatives for IBD because of their ability to modulate the gut microbiota, reinforce intestinal barrier integrity, and regulate immune responses. However, their clinical translation is hampered by challenges within the harsh gastrointestinal milieu, including low viability, poor colonization, and insufficient target specificity. This review focuses on the engineering of probiotics designed to overcome these limitations for IBD management. We outline the therapeutic potential and mechanisms of action of probiotics in IBD, with a critical emphasis on discrepancies between preclinical and clinical observations. We subsequently discuss the drawbacks of conventional probiotic therapies, highlighting gaps between *in vitro* efficacy and *in vivo* performance. We then highlight cutting-edge engineering strategies, encompassing advanced encapsulation techniques, genetic engineering approaches, novel delivery systems, and molecular-targeting modifications, with quantitative comparisons of their advantages, limitations, and translational potential. The application of these engineered probiotics specifically in UC and CD treatment is explored, with detailed analyses of preclinical models and clinical trials. We also address personalized interventions tailored to individual gut microbiome profiles. Despite significant promise, critical challenges remain, including long-term safety, stability, and accurate prediction of therapeutic responses for engineered probiotics in IBD. Nevertheless, with ongoing advancements in gene editing, synthetic biology, and microbial safety engineering, engineered probiotics represent a promising direction in IBD therapy that will enable more precise, effective, and personalized treatment modalities, provided that safety, reproducibility, and regulatory compliance are prioritized.

## 1 Introduction

Inflammatory bowel disease (IBD) is a chronic, relapsing inflammatory disorder of the gastrointestinal tract with significant geographical variations in global distribution. Canada has the highest prevalence of IBD (approximately 0.7%), followed by the United States (0.3%) and Europe (0.2%) (Shah and Itzkowitz, 2022; Wijnands et al., 2021). In the early 20th century, IBD primarily occurred in developed Western countries. However, with accelerated industrialization and urbanization and the adoption of Western lifestyles, the disease has rapidly spread to emerging industrialized regions (e.g., Asia, South America, and the Middle East), with sharp increases in its incidence and prevalence, making it a global public health concern (Aniwan et al., 2022; Caron et al., 2024; Wan et al., 2025).

### 1.1 Literature search strategy

To ensure comprehensiveness and rigor, a structured literature retrieval process was implemented in this review. We searched the PubMed, Web of Science, and Scopus databases using the following keywords: (“engineered probiotics” OR “genetically modified probiotics” OR “encapsulated probiotics”) AND (“inflammatory bowel disease” OR “ulcerative colitis” OR “Crohn’s disease”) AND (“mechanism” OR “delivery” OR “precision medicine” OR “clinical trial” OR “biosafety”). The retrieval period was limited to 2010–2025 to focus on recent advancements, with additional inclusion of foundational studies (e.g., Steidler et al., 2000) for context. We excluded preprint articles, conference abstracts, and studies with unclear methodologies or irrelevant endpoints. A total of 247 articles were initially screened, and 189 articles were included after full-text review—this narrative review integrates mechanistic, preclinical, clinical, and regulatory evidence to provide a balanced perspective.

### 1.2 Current challenges in IBD management

Currently, the clinical management of IBD primarily involves pharmaceutical therapies and surgical interventions (Agrawal et al., 2021; Muzammil et al., 2023; Liu et al., 2024). Commonly used drugs include aminosalicylates, corticosteroids, immunosuppressants, and biological agents. However, these drugs generally have problems such as low specificity, high toxicity and significant adverse reactions (Bruner et al., 2023; Singh et al., 2024). Approximately 15% of IBD patients worldwide require surgical intervention because of poor drug efficacy, but surgery is associated with high risks and long-term disability (Baumgart and Le-Berre, 2021; Villablanca et al., 2022).

### 1.3 Rationale for engineered probiotics in IBD

**Figure 1:**
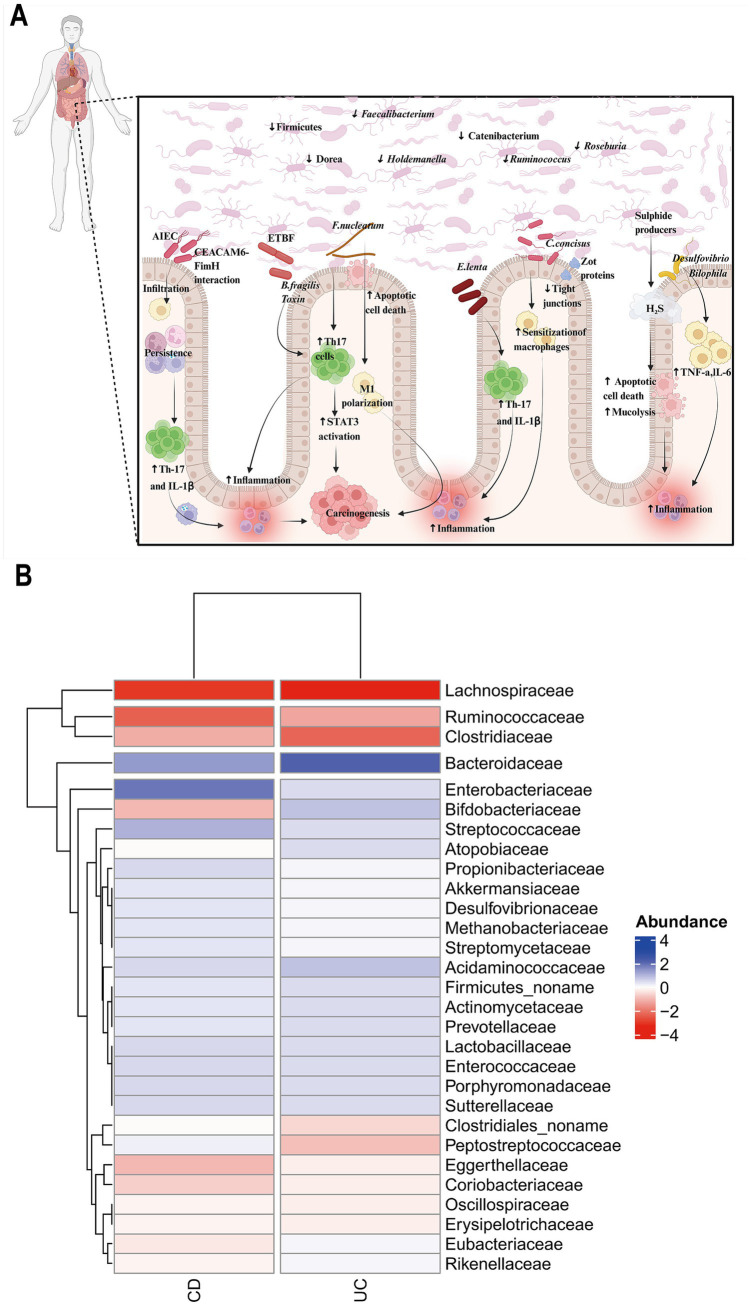
Intestinal microbial dysbiosis in IBD. **(A)** It is manifested primarily by a reduction in the abundance of beneficial bacteria (*Firmicutes*, *Dorea*, *Faecalibacterium*, *Holdemanella*, *Catenibacterium*, *Roseburia*, and *Ruminococcus*) and an increase in the abundance of harmful bacteria (AIEC, ETBF, *Fusobacterium nucleatum*, *Eggerthella lenta*, *Campylobacter concisus*, and sulfide producers). These changes disrupt the intestinal barrier, activate excessive immune responses, and exacerbate inflammation. AIEC: Adherent-invasive *E. coli*; ETBF: Enterotoxigenic *Bacteroides fragilis*; SCFAs: Short-chain fatty acids. **(B)** Heatmap of the relative abundance of gut bacterial changes in patients with CD and UC. It displays the relative abundance of gut bacterial species with increased (red) and decreased (blue) abundance [data from Arnau et al. (2018)]. Created by Biorender.

**Figure 2:**
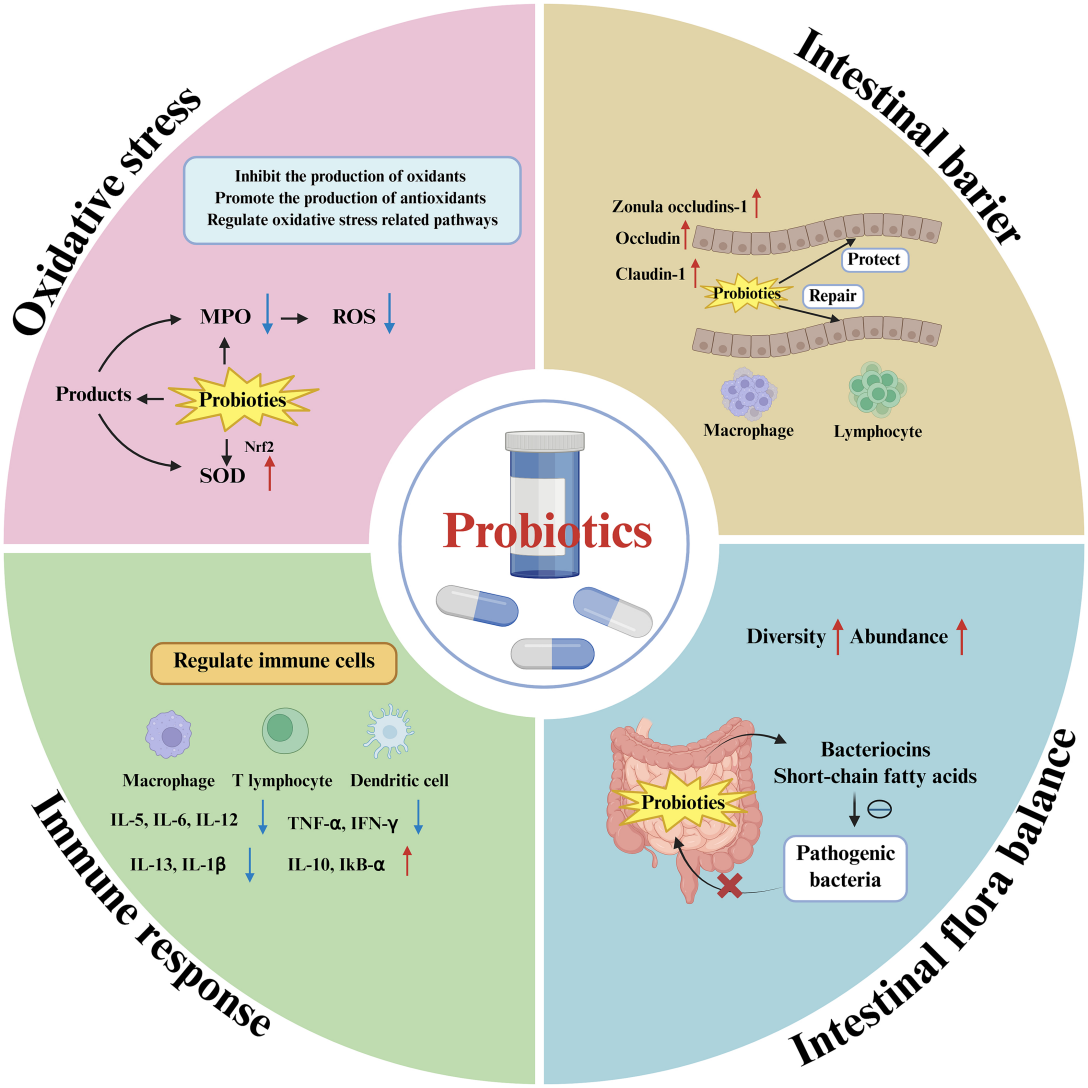
Probiotics alleviate or treat IBD by repairing the intestinal barrier, regulating the balance of the intestinal microbiota, reducing oxidative stress, and modulating intestinal immune responses. Antioxidant axis: secreted factors inhibit oxidant generation and promote endogenous antioxidant (superoxide dismutase, SOD) generation via Nrf2 activation, lowering mucosal ROS. Intestinal barrier repair: bacterial products increase the expression of the tight junction proteins zonula occludens-1 (ZO-1), occludin, and claudin-1, restoring epithelial integrity. Pathogen exclusion and microbiota reshaping: targeted bacteriocin production inhibits pathobionts, while SCFA-mediated pH reduction and niche competition increase overall microbial diversity and abundance; probiotics orchestrate immunomodulatory circuits that bias immune cells toward an anti-inflammatory phenotype, selectively upregulating anti-inflammatory cytokine expression while downregulating proinflammatory mediator expression to re-establish and maintain immune homeostasis. Created by Biorender.

Intestinal microbial dysbiosis plays a key role in IBD pathogenesis (Figure 1) (Rodrigues et al., 2022; Roy and Dhaneshwar, 2023). IBD patients usually have an imbalance in the composition of their intestinal microbiota, which is specifically manifested by a decrease in the number of beneficial bacteria and an increase in the number of harmful bacteria (Table 1). Probiotics can effectively inhibit the growth of pathogenic bacteria by competing for nutrients and adhesion sites. Moreover, the organic acids produced by probiotics can reduce the intestinal pH, thereby inhibiting the proliferation of pathogenic bacteria such as *Escherichia coli* (*E. coli*) and *Salmonella*. Probiotics can also increase the expression of tight junction proteins between intestinal epithelial cells and stimulate the secretion of mucin, thereby strengthening the physical, chemical and immune barriers and consolidating the microbial barrier (Figure 2). In terms of immune regulation, probiotics can promote the secretion of anti-inflammatory cytokines and inhibit that of proinflammatory factors, thereby alleviating the chronic inflammation caused by an excessive immune response (Xu et al., 2022; Zhao et al., 2022; Chen Y. et al., 2025; Chen Z. L. et al., 2025). Therefore, through various mechanisms, such as regulating intestinal flora balance, enhancing intestinal barrier function and modulating the immune response, probiotics have shown great potential and broad prospects in the treatment of IBD.

**Table 1:** Microbiota in inflammatory bowel disease: mechanisms of disease and therapeutic opportunities.

Bacteria	Dysbiosis in IBD	Mechanisms of pathogenesis/therapeutic benefit	References
*E. coli* (adherent and invasive)	Increased	*E. coli* adheres to and invades intestinal epithelial cells with high affinity, thereby surviving continuously in cells and evading clearance, activating excessive immune responses, destroying the integrity of the intestinal barrier, and simultaneously achieving dominant colonization by taking advantage of microbial imbalance. These changes ultimately lead to the initiation, amplification, and persistence of intestinal inflammation.	Ott et al. (2008) and Qiu et al. (2022)
*Klebsiella*	Increased	*Klebsiella* may participate in the occurrence and development of intestinal inflammation by stimulating intestinal immune responses, disrupting the intestinal barrier, or synergizing with other pathogenic bacteria, although the specific mechanisms remain under intensive investigation.	Kharaghani et al. (2023)
*Bacterooides fragilis* (*B. fragilis*)	Increased	The change in the abundance of *B. fragilis* in the intestines of IBD patients affects the balance of the gut microbiota. *B. fragilis* may damage the intestinal mucosal barrier through its metabolites, increasing the intestinal permeability. This allows harmful substances to easily enter the tissues, triggering an inflammatory response and promoting the development of IBD. Additionally, *B. fragilis* can regulate the immune response by secreting various substances, thus influencing the progression of IBD.	Tomkovich & Jobin (2015)
*Ruminococcus gnavus* (*R. gnavus*)	Increased	Excessive *R. gnavus* proliferation in the intestine may inhibit the production of IL-10 while promoting the release of pro-inflammatory factors such as IL-17, leading to the exacerbation of intestinal inflammation. *R. gnavus* can also activate immune cells, which release a large number of inflammatory mediators, further recruiting more immune cells to the inflamed site and forming an inflammatory cascade reaction, resulting in the damage and destruction of intestinal tissues.	Buttimer et al. (2023) and Hong et al. (2024)
*Clostridium perfringens* (*C. perfringens*)	Increased	*C. perfringens* directly attacks intestinal epithelial cells by secreting a variety of toxins, leading to cell necrosis or apoptosis. For instance, the toxic effects of toxins such as alpha toxin and beta toxin on cells impair the structure and function of epithelial cells, disrupt the tight junction structure between intestinal epithelial cells, and increase intestinal permeability.	Kuo et al. (2024)
*Clostridium tertium* (*C. tertium*)	Increased	*C. tertium* may disrupt the integrity and tight junctions of epithelial cells by secreting toxins or directly interacting with intestinal epithelial cells. Additionally, *C. tertium* may compete with beneficial bacteria for ecological niches and nutrients, thereby inhibiting the growth and reproduction of beneficial bacteria.	Li et al. (2021)
*Faecalibacterium Prausnitzii* (*F. prausnitzii*)	Decreased	*F. prausnitzii* can increase the number and activity of Tregs and promote Tregs to secrete anti-inflammatory cytokines such as IL-10. *F. prausnitzii* may secrete certain bioactive substances to promote the proliferation and differentiation of intestinal epithelial cells, thereby accelerating the repair of damaged mucosa. Additionally, *F. prausnitzii* can alleviate colonic inflammation by inhibiting the NLRP3 inflammasome and its downstream effector molecules.	Varela et al. (2013), Wang X. L. et al. (2025), and Wang Z. Y. et al. (2025)
*Roseburia intestinalis* (*R. intestinalis*)	Decreased	*R. intestinalis* can inhibit the development of IBD by increasing the differentiation of anti-inflammatory Tregs. *R. intestinalis* may alleviate intestinal inflammation by regulating the immune microenvironment and inhibiting the aggregation and activation of these pro-inflammatory cells. Additionally, *R. intestinalis* may indirectly affect the disease progression of IBD through the production of short-chain fatty acids.	Alizadeh et al. (2025)
*Bifidobacterium*	Decreased	Supplementation with *Bifidobacterium* can increase the number of beneficial bacteria in the intestine and improve the intestinal microecological environment. *Bifidobacterium* can exert anti-inflammatory effects by regulating the secretion of cytokines by immune cells. *Bifidobacterium* produces metabolites such as short-chain fatty acids, which promote the growth and repair of intestinal epithelial cells.	Li M. F. et al. (2024) and Li M. M. et al. (2024)
*Lactobacillus*	Decreased	*Lactobacillus* restores the Th1/Th2 balance to a normal state by regulating the secretion of cytokines, thereby alleviating inflammatory responses. *Lactobacillus* may influence the distribution and function of tight junction proteins by regulating intracellular signaling pathways, thus enhancing the connections between intestinal epithelial cells. Additionally, *Lactobacillus* can produce metabolites such as SCFAs, which can provide energy for intestinal epithelial cells, regulate intestinal pH, and inhibit inflammatory responses, among other functions.	Li et al. (2023)
*Akkermansia muciniphila* (*A. muciniphila*)	Decreased	*A. muciniphila* contributes to restoring and enhancing the integrity of the mucus layer by promoting the production of mucin, thereby exerting a protective effect on the intestine. *A. muciniphila* may repair and strengthen the intestinal epithelial barrier by regulating the expression of tight junction proteins, thus alleviating inflammatory responses. *A. muciniphila* maintains the integrity of the intestinal barrier by reducing the expression of pro-inflammatory cytokines and the infiltration of inflammatory cells, as well as increasing the expression of anti-inflammatory cytokines and the differentiation of Tregs, thereby alleviating colonic damage and colitis.	Aja et al., 2025, Liu J. et al. (2022), and Liu Y. J. et al. (2022)
*Eubacterium rectale* (*E. rectale*)	Decreased	A reduction in the abundance of bacteria such as *E. rectale* may impair the normal function of the intestinal mucus layer, weakening its ability to resist pathogens and thereby increasing the risk of intestinal inflammation. A decrease in the abundance of *E. rectale* may disrupt immune homeostasis, leading to excessive activation of Th1 immune responses and exacerbating intestinal inflammation. Changes in the quantity or function of *E. rectale* may result in insufficient production of butyrate, depriving the intestine of the protective effects of butyrate—such as the inhibitory effects on inflammation and protective effects on intestinal barrier function—thereby promoting the development of IBD.	Pittayanon et al. (2020)
Veillonellaceae	Increased	*Veillonella* may affect the expression and release of cytokines, leading to an imbalance between pro-inflammatory cytokines and anti-inflammatory cytokines. *Veillonella* may impair the function of intestinal mucus-secreting cells or directly degrade mucin components in the mucus layer, resulting in thinning of the mucus layer and damage to its integrity.	Tursi et al. (2025)
*Collinsella aerofaciens* (*C. aerofaciens*)	Decreased	*C. aerofaciens* may be involved in the pathogenesis and development of inflammatory bowel disease through multiple aspects, such as regulating the structure of intestinal flora, affecting intestinal barrier function, and interfering with host immune regulation and butyrate production.	Quagliariello et al. (2020)
*Lactobacillus* GG (LGG)	Decreased	LGG may influence the differentiation direction of Th cells by regulating the function of antigen-presenting cells such as dendritic cells, thereby correcting the Th1/Th2 imbalance and alleviating inflammatory responses. The ROS induced by LGG can oxidize the key regulatory enzyme Ubc12, inactivating it, thus inhibiting the phosphorylation of IκB, preventing NF-κB from entering the nucleus, reducing the transcription and expression of inflammatory factors, and further alleviating intestinal inflammation. Additionally, LGG can promote the secretion of mucin by intestinal epithelial cells, increasing the thickness and viscosity of the mucus layer and thereby enhancing the physical barrier function of the intestine.	Zocco et al. (2006) and Zhai et al. (2024)
*Bacillus subtilis* (*B. subtilis*)	Decreased	*B. subtilis* can upregulate the expression of tight junction proteins and enhance intestinal barrier function. It may regulate the function of intestinal goblet cells, promote mucus secretion, increase the thickness of the mucus layer, and thereby strengthen the protection of intestinal epithelium. Additionally, *B. subtilis* can inhibit the expression of pro-inflammatory cytokines and promote the production of anti-inflammatory cytokines, which helps balance the body’s immune response and alleviate intestinal inflammation.	Liu S. et al. (2021) and Liu W. et al. (2021)
*Fusobacterium nucleatum* (*F. nucleatum*)	Increased	*F. nucleatum* can promote the apoptosis of intestinal epithelial cells, destroy tight junction proteins, exacerbate intestinal inflammatory responses, and induce intestinal flora dysbiosis.	Chen et al. (2020)
*Enterococcus faecium* (*E. faecium*)	Increased	ROS produced by *E. faecium* cause cellular oxidative stress damage, disrupt the intestinal mucosal barrier, and trigger inflammatory responses. Abnormal proliferation of *E. faecium* or certain substances produced by it may affect intestinal barrier function. As a part of the intestinal flora, the abnormal interaction between *E. faecium* and the host immune system may lead to immune imbalance, thereby inducing or exacerbating inflammatory bowel disease.	Seishima et al. (2019)
Firmicutes	Decreased	Phylum Firmicutes influences the disease mechanisms of inflammatory bowel disease through multiple pathways such as intestinal barrier function, immune regulation, bile acid metabolism, production of short-chain fatty acids, and oxidative stress.	Li et al. (2016)

Dysbiosis patterns may vary between UC and CD (e.g., *F. nucleatum* is more strongly associated with CD), and interindividual heterogeneity is common.

However, traditional probiotics still face many challenges in the treatment of IBD, such as low survival rate, poor colonization efficiency and insufficient targeting. To overcome these problems, increasing the survival rate and improving the colonization ability of probiotics through engineering modification and enhancing their ability to accurately recognize inflammatory signals and achieve targeted localization are highly important. This approach is expected to not only significantly strengthen the therapeutic effect, achieve precise treatment, and prevent disease recurrence but also promote the development of personalized medicine, offering comprehensive health benefits to several patients.

## 2 Therapeutic effects of probiotics on IBD and the related molecular mechanisms

### 2.1 Strategies for restoring the gut microbiota equilibrium

#### 2.1.1 Restoration of gut microbiota diversity

In IBD patients, the diversity of the gut microbiota is generally reduced (Leibovitzh et al., 2022). Probiotics can reduce the abundance of harmful bacteria by competing for nutrients and spatial niches, thereby promoting the proliferation of beneficial bacteria and restoring microbiota diversity (Zhang and Wang, 2024; Yuan et al., 2024). For instance, species from the genus Bifidobacterium can utilize oligosaccharides and other gut substances to grow and reproduce, simultaneously producing organic acids that reduce the intestinal pH. This acidic environment suppresses the growth of pathogenic bacteria such as *E. coli* and promotes the growth of beneficial bacteria, including bifidobacteria and lactobacilli, ultimately reestablishing gut microbial diversity. Therefore, key probiotic strains that can enhance the diversity restoration effects of endogenous commensal microbiota should be screened, and prebiotic–probiotic combinations targeting IBD-specific microbial deficiencies should be developed.

#### 2.1.2 Strategies for altering the microbiota structure

The structure of the gut microbiota is altered in IBD patients, characterized by an imbalance in the ratio of Firmicutes to Bacteroidetes (Lloyd-Price et al., 2019; Liu S. et al., 2021; Liu W. et al., 2021). Probiotics can reverse this imbalance. Probiotics can aid in the restoration of the microbiota structure through strain-specific targeting of microbial taxa, with recent research (2024–2025) revealing the mechanisms through which probiotics affect niche competition and metabolic crosstalk.

Recent studies on Lactobacillus-mediated Firmicutes enrichment (Stojanov et al., 2020) highlight the following strategies: *Bifidobacterium longum* subsp. infantis BLI-02 increases its own fucosidase activity to utilize host-derived fucosylated mucins, outcompeting mucin-degrading Bacteroidetes (e.g., *Bacteroides fragilis*) and restoring the F/B ratio (Kuo et al., 2023); and *Enterococcus faecalis* SF68 adheres to intestinal epithelial cells via adhesin efaA, blocking adhesion sites for pathogenic Proteobacteria while it secretes succinate to promote Firmicutes colonization (Abdi et al., 2021; Holzapfel et al., 2023). Notably, *Lactobacillus plantarum* WCFS1 modulates quorum sensing in pathobionts—its secreted autoinducer-2 (AI-2) analogs inhibit *E. coli* biofilm formation, reducing the abundance of Proteobacteria without disrupting that of commensal bacteria (Kim et al., 2024). These structural adjustments normalize microbial–epithelial crosstalk, alleviating IBD-associated dysbiosis. Therefore, future studies should perform the mapping of strain-specific effects on microbial metabolic networks to predict normalization of fecal/bacterial ratios and develop precision probiotics on the basis of baseline microbiome profiles.

### 2.2 Strategies for fortifying the intestinal epithelial barrier

#### 2.2.1 Promotion of mucus layer secretion

The intestinal mucus layer—primarily composed of MUC2 and glycosylated glycoproteins—forms a physical and chemical barrier that sequesters pathogens and limits antigen access to epithelial cells. In IBD, reduced MUC2 expression and aberrant glycosylation decrease the thickness of the mucus layer, exacerbating epithelial exposure to luminal insults (Li M. F. et al., 2024; Li M. M. et al., 2024). Probiotics enhance mucus barrier function through conserved and strain-specific signaling, with recent studies (2024–2025) clarifying the crosstalk mechanism.

**Figure 3:**
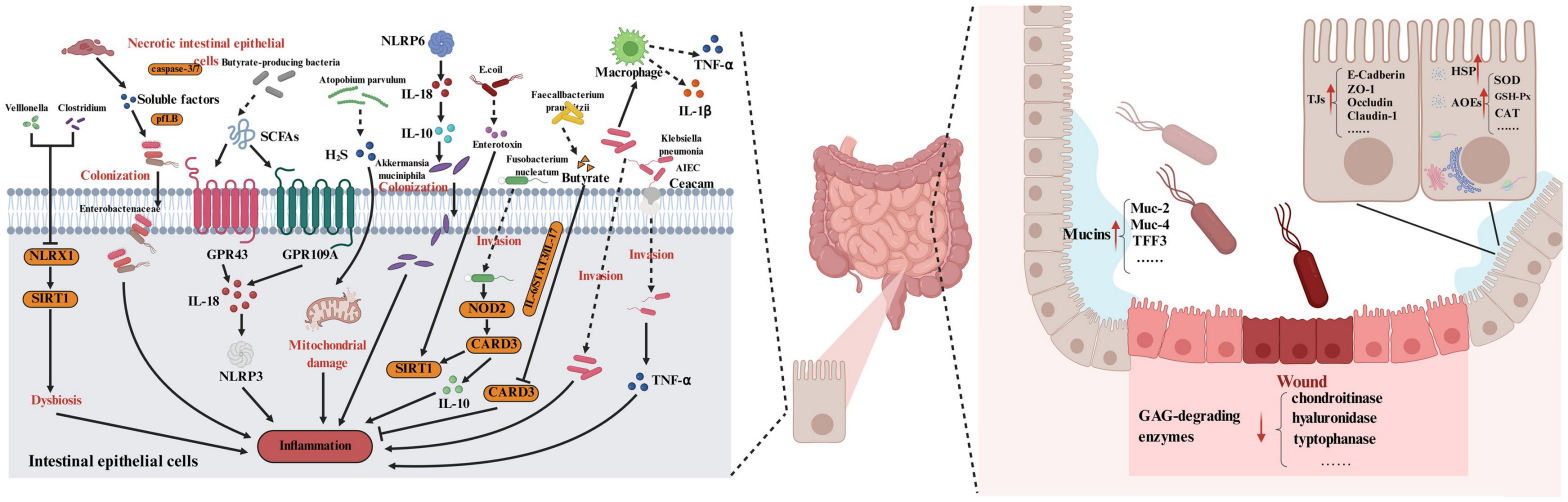
Mechanism underlying the barrier-protective effect of probiotics in IBD. Probiotics enhance the integrity of the mucous barrier by upregulating protective mucin expression. Simultaneously, they reinforce the cellular barrier through effects on intestinal epithelial tight junctions and the expression levels of intracellular protective proteins. Additionally, probiotics maintain the intestinal immune barrier by modulating immune cell populations and the expression of inflammatory mediators. Created by Biorender.

In addition to *Lactobacillus reuteri*-mediated activation of epithelial pathways (Li M. F. et al., 2024; Li M. M. et al., 2024), diverse strains target goblet cell function: *Bifidobacterium breve* YIT 12272 engages TLR2 on goblet cells to activate the p38 MAPK pathway, increasing MUC2 transcription (Wang X. L. et al., 2025; Wang Z. Y. et al., 2025); *Lactobacillus gasseri* ATCC 33323 stimulates mucin glycosylation by upregulating galactosyltransferase (β4GalT1), enhancing mucus resistance to bacterial proteases (Chen Y. et al., 2025; Chen Z. L. et al., 2025). Probiotic-derived metabolites also play key roles: SCFAs from *Clostridium butyricum* inhibit histone deacetylase 1 (HDAC1) in goblet cells, relieving the transcriptional repression of MUC5AC (a minor but functionally critical mucin) (Huang et al., 2016). Notably, the increase in mucus layer thickness by probiotics reciprocally supports microbiota balance—thicker mucus provides a niche for commensals, reinforcing a “barrier-microbiota” feed-forward loop. For instance, *Lactobacillus reuteri* can activate intracellular signaling pathways within epithelial cells, promoting the synthesis and secretion of mucin MUC2. This strengthening of the mucus layer effectively protects the intestinal mucosa and mitigates tissue damage in IBD patients (Figure 3) (Li M. F. et al., 2024; Li M. M. et al., 2024). Therefore, future research directions include deciphering strain-specific regulatory mechanisms of mucin glycosylation to counteract the glycosylation defects associated with inflammatory bowel disease; exploring the interactions among probiotics, intestinal glial cells, and goblet cells; and developing mucin-mimetic prebiotics to synergize with probiotics to restore the mucus layer.

#### 2.2.2 Strategies for strengthening epithelial tight junctions

Tight junctions (TJs)—composed of transmembrane proteins and cytoplasmic scaffolding proteins—regulate paracellular permeability. In IBD, reduced TJ protein expression/assembly increases permeability, enabling luminal antigens to trigger mucosal inflammation (Deng et al., 2024). Probiotics reinforce TJs through convergent signaling pathways, with recent studies (2024–2025) highlighting strain diversity and posttranslational regulation.

In terms of *Lactobacillus acidophilus*-mediated PI3K/Akt activation (Deng et al., 2024), other strains act via complementary mechanisms: *Lactobacillus rhamnosus* GG (LGG) secretes the pili protein SpaC, which binds epithelial E-cadherin to activate AMPK, promoting the phosphorylation of occludin at Ser408 and its membrane localization (Zhang and Wang, 2024), and *Bifidobacterium lactis* BB-12 downregulates the expression of miR-200c, a microRNA that targets claudin-1 mRNA, thereby increasing claudin-1 protein levels (Splichalova et al., 2021). Probiotic EVs also contribute—*Streptococcus thermophilus* EVs carry miR-148a, which inhibits the ubiquitin ligase *β*-TrCP, preventing ZO-2 degradation (Liu et al., 2024). These coordinated effects reduce permeability, limiting antigen influx and inflammation (Figure 3) (Deng et al., 2024). Therefore, future research directions may include mapping cross-regulatory networks between tight junction regulatory pathways to identify master regulators that can be targeted by multistrain probiotics; genetically engineering probiotics to overexpress factors that stabilize tight junctions; and investigating how microbiota metabolites synergize with probiotics to maintain tight junction protein function.

### 2.3 Immune reprogramming strategies

IBD is driven by immune dysregulation, hyperactivation of proinflammatory pathways and insufficient anti-inflammatory signals (Ruiz et al., 2017; Gavzy et al., 2023). Probiotics alleviate these pathological changes through strain-specific interactions with innate and adaptive immune cells, with recent studies (2024–2025) revealing their broad mechanisms.

Beyond Bifidobacteria-mediated dendritic cell (DC) activation (Gavzy et al., 2023), diverse probiotics target multiple immune checkpoints: *Lactobacillus plantarum* WCFS1 activates NOD2 in macrophages to suppress NF-κB p65 translocation, reducing TNF-*α*/IL-6 secretion (Ohgi et al., 2024), and *Bifidobacterium adolescentis* induces DCs to secrete IL-27, which promotes Treg differentiation by upregulating Foxp3 and inhibiting ROR*γ*t (Th17 master regulator) expression. Additionally, probiotic-derived indole-3-pyruvate modulates mucosal-associated invariant T (MAIT) cells, shifting their cytokine profile from that of IFN-γ to that of IL-10 (Wu et al., 2022).

## 3 Limitations of traditional probiotics in IBD treatment

### 3.1 Low probiotic survival in the gastrointestinal microenvironment

#### 3.1.1 Acidic pH in the stomach

**Figure 4:**
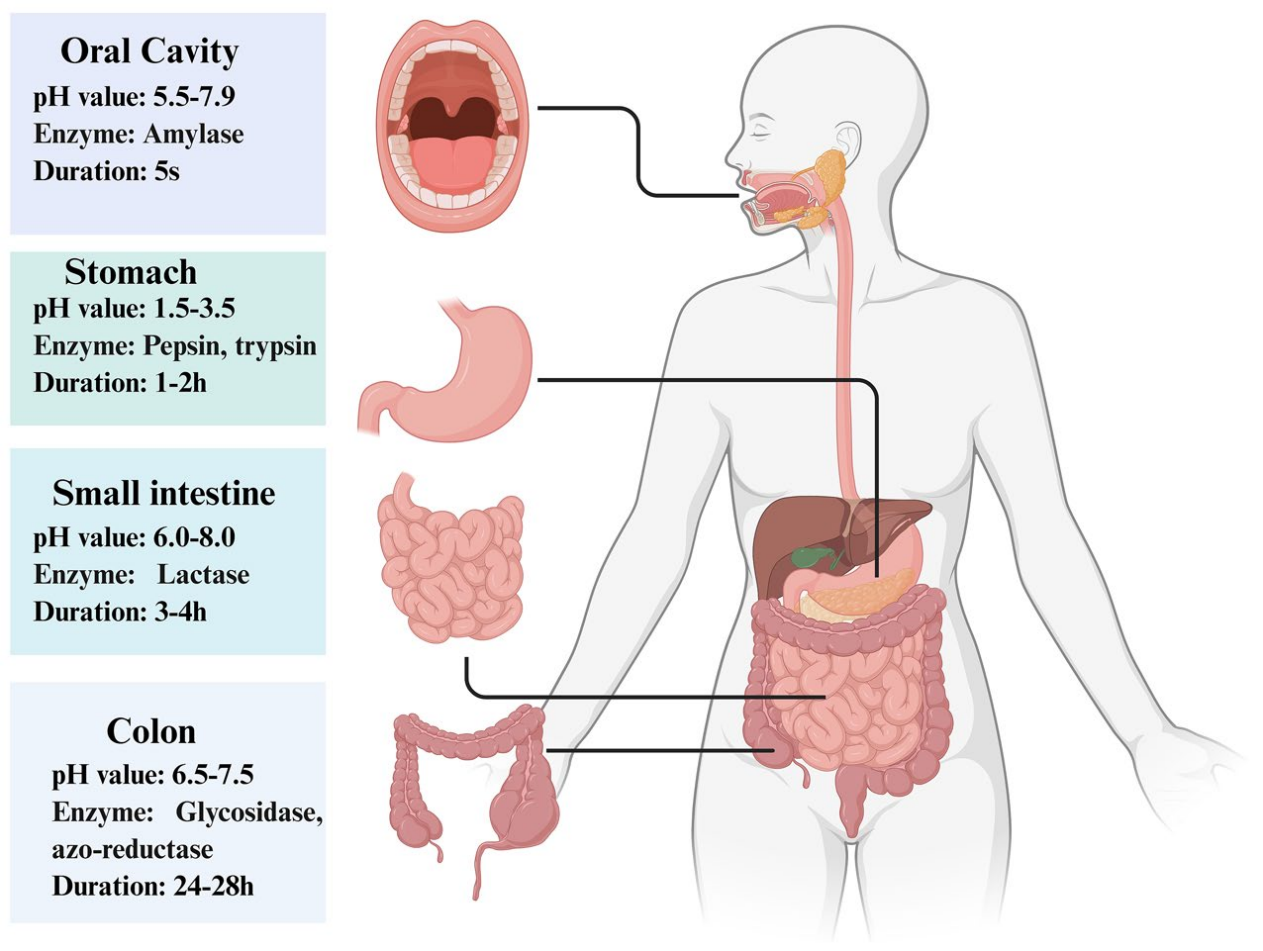
Characterization of the digestive environments in four key segments of the human digestive system, namely, the oral cavity, stomach, small intestine, and colon, including their respective pH ranges, major digestive enzymes, and food transit times. The oral cavity has a pH range of approximately 5.5–7.9 and contains amylase, with a relatively short food transit time. The stomach is highly acidic, with a pH range of approximately 1.5–3.5, and is equipped with major digestive enzymes such as pepsin and trypsin. In the stomach, food is triturated and mixed with gastric acid and pepsin, undergoing preliminary digestion into “chyme.” The small intestine is weakly acidic to weakly alkaline (pH 6.0–8.0) and harbors a variety of digestive enzymes. As the primary site for digestion and absorption, the vast majority of nutrients are completely decomposed and absorbed here, with a digestion duration of 3–4 h. The colon is weakly acidic to neutral (pH 6.5–7.5), and the major enzymes involved in this process include glycosidase and azoreductase, which are secreted by the intestinal flora to decompose undigested substances. Food residues reside here for an extended period, completing the final metabolic processes and feces formation. Created by Biorender.

The gastric environment is highly acidic, typically ranging from pH 1.5 to 3.5 (Figure 4). This extreme acidity significantly threatens the survival of probiotics (Hlaing et al., 2020; Koga, 2022; Cai et al., 2025). As environmentally sensitive microorganisms, probiotics are vulnerable to damage under acidic conditions, particularly with respect to their cell membrane integrity. When the cell membrane, as a critical barrier, is compromised, intracellular contents may leak, thereby disrupting normal metabolic activities. Additionally, essential enzyme systems responsible for energy metabolism can become denatured and dysfunctional in an acidic milieu, preventing cells from producing sufficient energy to sustain life. Furthermore, the acidic environment may induce structural alterations in probiotic DNA, adversely affecting gene expression and interfering with its growth, reproduction, and specific biological functions. Consequently, prolonged exposure to such highly acidic conditions leads to a sharp decline in the number of viable probiotic cells, resulting in substantial cell death before the cells reach the intestine and thus limiting their therapeutic efficacy.

#### 3.1.2 Threat of bile salts in the small intestine

Once probiotics manage to traverse the stomach, they encounter another significant challenge posed by bile salts within the small intestine. Bile salts, which are secreted by the liver and stored in the gallbladder, are essential for fat digestion and absorption in the small intestine (Hu et al., 2018; Luo et al., 2022). However, bile salts are a double-edged sword for probiotics. Their amphipathic structure, possessing both hydrophilic and hydrophobic ends, enables them to interact with the probiotic cell membrane, disrupting the lipid bilayer and increasing membrane permeability (Gao et al., 2022). *In vitro* studies revealed that 0.5% (w/v) bile salt increased the membrane permeability of *L. rhamnosus* GG by 72 ± 4.6%, as measured by propidium iodide (PI) staining, whereas *L. casei* 393 exhibited only a 28 ± 3.1% increase in membrane permeability because of increased bile salt hydrolase (BSH) activity. BSH-positive strains can deconjugate bile salts into less toxic forms, with *L. casei* 393 achieving a 52 ± 3.8% deconjugation rate within 4 h, compared with 17 ± 2.5% in BSH-deficient *L. plantarum* Δbsh (Luo et al., 2022). Enhanced membrane permeability disturbs the balance between intracellular and extracellular substances, leading to the loss of essential ions and small molecules while permitting harmful external substances to penetrate the cells, further compromising cellular functions. Additionally, bile salts can interfere with probiotic metabolic pathways, suppressing their growth and proliferation. In bile-containing environments, the probiotic growth rate significantly decreases, and cell division and proliferation are inhibited, thereby markedly decreasing the number of viable probiotic cells.

#### 3.1.3 Other adverse factors

Apart from gastric acidity and the presence of bile salts in the small intestine, additional factors within the gastrointestinal tract adversely affect probiotic survival. For instance, digestive enzymes such as pepsin in the stomach and trypsin in the pancreas can damage the digestion of food by probiotics (Sorbara and Pamer, 2019; Luo et al., 2022; Du et al., 2024). These enzymes specifically recognize and hydrolyze proteins, and since probiotic cell walls and membranes contain substantial amounts of protein components, they are susceptible to enzymatic degradation, leading to cell death. The survival rate of *B. bifidum* Bb-02 was 78 ± 4.3% after 1 h of exposure to 0.8 mg/mL pepsin, whereas that of *L. rhamnosus* GR-1 was 58 ± 3.5% because of the presence of a thicker peptidoglycan layer. Furthermore, the complex microbial community within the gut is another critical factor. The intestine naturally harbors numerous symbiotic bacteria that have evolved to form relatively stable ecosystems. Upon entry into the intestine, exogenous probiotics must compete with these resident bacteria for nutrients and adhesion sites. Symbiotic bacteria may produce antimicrobial substances that hinder the growth and reproduction of probiotics, making it difficult for them to survive and colonize such a competitive environment and resulting in markedly reduced probiotic viability.

### 3.2 Suboptimal probiotic colonization efficacy

#### 3.2.1 Competition with the native gut microbiota for nutrients and adhesion sites

From a nutritional perspective, the resources available in the gut—including carbon and nitrogen sources, vitamins, and minerals essential for growth—are highly contested. Over evolutionary timescales, the native gut microbiota has adapted specifically to the nutritional environment of the intestine, developing specialized mechanisms for nutrient uptake and metabolism (Sorbara and Pamer, 2019). For example, certain native gut bacteria efficiently utilize specific polysaccharides as carbon sources by secreting enzymes that degrade these polysaccharides into absorbable monosaccharides, thereby gaining a competitive advantage in nutrient acquisition. In contrast, they struggle to compete effectively for nutrients against colonization and proliferation within the intestine (Jensen et al., 2023; Cherrak et al., 2024).

In terms of competition for adhesion sites, the intestinal mucosal surface provides limited adhesion spaces that are critical for microbial colonization in the gut (Freter et al., 1983; Caballero-Flores et al., 2023; Cherrak et al., 2024). Many members of the natural gut microbiota occupy these sites by strongly binding to specific receptors on the surface of intestinal epithelial cells via specialized surface adhesion factors such as pili, capsules, and similar structures. Such robust binding enables stable colonization despite intestinal peristalsis and the flushing effect of digestive fluids while also facilitating nutrient acquisition and resistance to external interference. When conventional probiotics attempt to colonize the intestinal mucosa, they must compete with the native microbiota that has already occupied these adhesion sites. Conventional probiotics often lack sufficient adhesion factors that are highly compatible with these sites or possess weaker adhesive capacities, making stable attachment to the intestinal mucosal surface challenging (Oliveira et al., 2020; Eberl et al., 2021). Even probiotics that can achieve temporary adhesion may frequently be displaced by competition with the native microbiota, preventing them from establishing stable populations within the gut. This dual competition for both nutrients and adhesion sites significantly hinders the establishment of a stable microbial community by conventional probiotics in the intestine, markedly reducing their colonization efficiency (Litvak et al., 2019; Osbelt et al., 2021). *Eubacterium rectale* binds to MUC2 via its surface lectin EreA, achieving an adhesion rate of 4.2 × 10^5^ CFU/cm^2^, whereas conventional probiotics such as *L. bulgaricus* 11,842 exhibit an adhesion rate of only 8.3 × 10^3^ CFU/cm^2^ (Caballero-Flores et al., 2023).

#### 3.2.2 Lack of specific colonization factors for some probiotics

Specific colonization factors are crucial for the long-term survival and functionality of probiotics within the gut (Chen Y. et al., 2025; Chen Z. L. et al., 2025). Certain probiotics possess specific surface proteins that recognize and bind specific receptors on the surface of intestinal epithelial cells, enabling strong adhesion between probiotics and epithelial cells (Cherrak et al., 2024; Zhang et al., 2025). This adhesion not only helps probiotics resist flushing by digestive fluids and intestinal peristalsis but also promotes effective interactions with host cells, thereby modulating the host immune response and intestinal barrier function. For example, the SpaC protein of *L. rhamnosus* GG mediates binding to MUC2, increasing resistance to intestinal peristalsis and digestive fluid flushing—its retention time in the colon is 3.2 ± 0.3 days, whereas it is 1.1 ± 0.1 days for SpaC-deficient mutants. Additionally, some colonization factors enable probiotics to form protective biofilms within the gut. Biofilms offer probiotics a relatively stable microenvironment, shielding them from harmful external factors and facilitating intercellular communication and synergistic effects among probiotics (Gao et al., 2025; Yao et al., 2025; Phùng et al., 2025; Yan et al., 2025). *Bifidobacterium longum* expresses the surface protein BlcA, which induces biofilm formation with a coverage rate of 65 ± 4.2% on intestinal epithelial cells, protecting the bacteria against bile salts and ROS (Chen Y. et al., 2025; Chen Z. L. et al., 2025).

However, certain conventional probiotics lack these specific colonization factors, resulting in numerous challenges in the gut. These probiotics tend to reside only transiently within the intestinal tract and are quickly expelled because of intestinal peristalsis and digestion. This phenomenon is due to the absence of an effective adhesion mechanism that enables them to attach firmly to the intestinal mucosal surface, making them vulnerable to removal by digestive fluids (Yan and Polk, 2021; Han et al., 2021). Furthermore, their inability to form stable biofilms renders them especially susceptible to harmful gastrointestinal conditions, such as stomach acid, bile salts, and reactive oxygen species. This transient colonization severely limits their ability to continuously modulate the gut microbiota and improve intestinal barrier function, significantly reducing their therapeutic efficacy in the treatment of IBD (Sang et al., 2025).

### 3.3 Insufficient targeting of probiotics

The inflamed intestinal regions in IBD patients exhibit unique pathological microenvironments, such as elevated levels of reactive oxygen species due to inflammation and alterations in the microbial community structure (Ashique et al., 2023; Muro et al., 2024). However, conventional probiotics lack the mechanisms required to recognize and respond to these specific microenvironmental signals. Upon entering the gastrointestinal tract, they are distributed randomly such that they are unable to actively locate and concentrate in inflamed regions. This nonspecific distribution leads to a significant number of probiotics failing to reach the inflamed regions where their effects are most needed. Instead, they often remain in healthy areas of the intestine, yet they exert minimal therapeutic effects on immune dysregulation and impaired barrier function at the inflamed regions. Owing to this insufficient localization, conventional probiotics struggle to effectively regulate the intestinal immune response, repair the damaged intestinal barrier, and mitigate gut microbiota dysbiosis (Eberl et al., 2021; Zhang et al., 2025; Sang et al., 2025). Nontargeted delivery may also result in unintended off-target effects. When probiotics are widely distributed across noninflamed regions, they can disrupt the normal physiological functions of the intestine. For example, excess probiotics could alter the microbial community balance in healthy intestinal areas, negatively affecting the interactions among symbiotic microorganisms. Such disturbances may trigger a series of adverse consequences, such as intestinal dysfunction, leading to symptoms such as abdominal pain and diarrhea in patients. Additionally, unnecessary interactions with normal intestinal tissues may provoke an excessive immune response against probiotics, increasing the risk of immune-related adverse reactions (Veiga et al., 2020). In the long term, these off-target effects may not only fail to treat IBD effectively but could impose additional risks and adverse impacts on the patient’s intestinal health.

## 4 Probiotic engineering strategies for IBD treatment

### 4.1 Encapsulation technology

**Figure 5:**
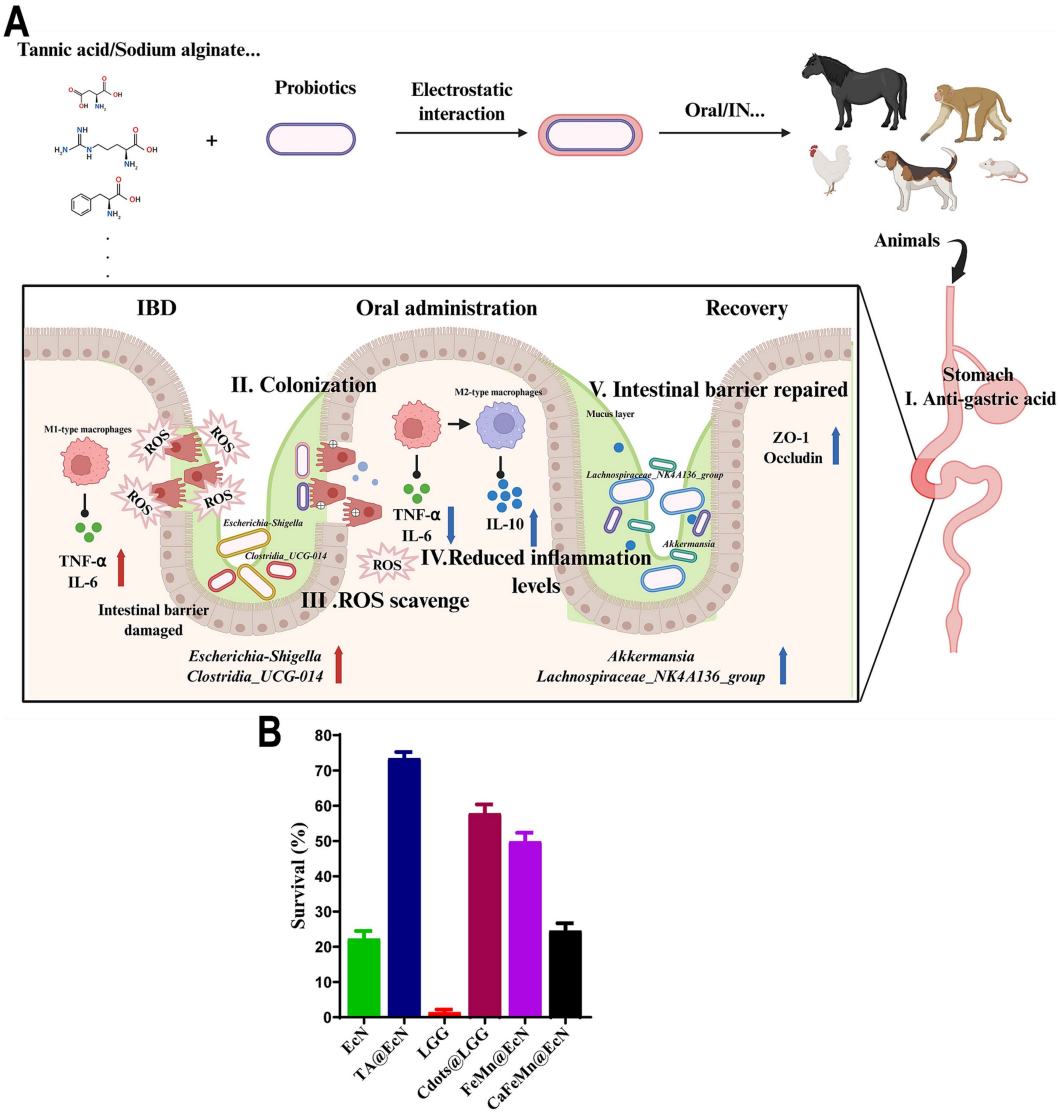
Encapsulation technology enhances the efficacy of probiotics in alleviating or treating IBD. **(A)** Probiotics encapsulated with polymeric materials such as tannic acid, sodium alginate, and chitosan can increase their tolerance to gastric acid and bile salts after oral administration, improve their intestinal colonization capacity, and increase their efficacy in alleviating or treating intestinal inflammation. **(B)** EcN, TA@EcN, LGG, Cdots@LGG, FeMn@EcN, and CaFeMn@EcN were exposed to simulated gastric fluid for 30 min, respectively. The survival rate of probiotics encapsulated with different nanomaterials was determined using the plate counting method. EcN: *E. coli* Nissel 1917; TA: tannic acid; Cdots@LGG: carbon dots are applied to modify *Lactobacillus rhamnosus* GG; FeMn@EcN: manganese chloride (MnCl_2_) was introduced before ferric chloride (FeCl_3_) into the EcN suspension, allowing a balanced electrostatic adsorption of both Mn^2+^ and Fe^3+^ ions onto the negatively charged bacterial surface. CaFeMn@EcN: FeMn@EcN was dispersed in a solution of polyvinylpyrrolidone (PVP) and calcium chloride (CaCl_2_), allowing Ca^2+^ ions to be adsorbed onto the bacterial surface (termed Ca^2+^FeMn@EcN) as the deposited PVP could capture Ca^2+^ ions by the complexation with the pyrrolidone pendant groups [data from Luo et al. (2023), Chen Y. et al. (2025), and Chen Z. L. et al. (2025)]. Created by Biorender.

Encapsulation technology serves as a core strategy to improve probiotic survival during oral administration. Probiotics face severe survival challenges from gastric acid and bile, which drastically reduce their viability before they reach the intestine. Encapsulation provides a protective barrier, enabling probiotics to safely transit the stomach and exert beneficial effects in the intestine (Figure 5) (Shanuke et al., 2025).

Two primary approaches are microencapsulation and nanoparticle encapsulation, which use polymer materials to enclose probiotics in tiny capsules or particles (Zhou et al., 2022; Hu et al., 2024). These materials include natural polymers and synthetic polymers. The sodium alginate–chitosan double-layer encapsulation method has been demonstrated to significantly increase the survival rate of probiotics, and *Lactobacillus rhamnosus* GG encapsulation via this technique markedly increases its survival rate in simulated gastrointestinal fluids (Shanuke et al., 2025). This method leverages the gelation properties of sodium alginate and the biocompatibility of chitosan to form a protective film that minimizes probiotic exposure to gastric acid and bile (Oberoi et al., 2021).

Another promising approach is the deployable physical containment strategy (DEPCOS), an alginate-based hydrogel encapsulation technology. It enhances probiotic survival in acidic environments (pH 4) and prolongs intestinal residence time, thereby improving therapeutic efficacy (Tang et al., 2021). Its unique hydrogel structure remains stable in gastric acid and gradually releases probiotics in the intestine.

Compared with genetic engineering, encapsulation offers a nongenetically modified (non-GMO) route, avoiding safety concerns and regulatory barriers associated with transgenic microorganisms, which is critical for clinical translation in regions with strict GMO oversight (Shanuke et al., 2025). It reliably protects probiotics from gastrointestinal stressors with well-documented *in vitro*/*in vivo* improvements in probiotic survival, outperforming unmodified probiotics and early delivery systems in terms of transit stability. Its broad compatibility with natural and synthetic polymers allows for the customization of strain-specific needs and clinical goals. Additionally, encapsulation integrates seamlessly with delivery systems to synergistically increase survival and targeting, offering flexibility that is lacking in standalone genetic engineering.

Unlike genetic engineering, encapsulation cannot tailor the intrinsic functions of probiotics—its role is passive protection rather than functional enhancement. Balancing high encapsulation efficiency with precise controlled release remains a bottleneck; overly dense coatings may preserve viability but hinder intestinal release, while porous structures compromise stress resistance. Compared with advanced responsive delivery systems, traditional encapsulation has lower targeting precision, often resulting in nonspecific release across the intestine rather than focusing on inflamed lesions. Potential material-induced immune responses and scalability limitations for large-scale production further constrain its utility, especially compared with the scalable fermentation of engineered probiotics.

### 4.2 Novel delivery systems

#### 4.2.1 Surface modification and targeting enhancement

**Figure 6:**
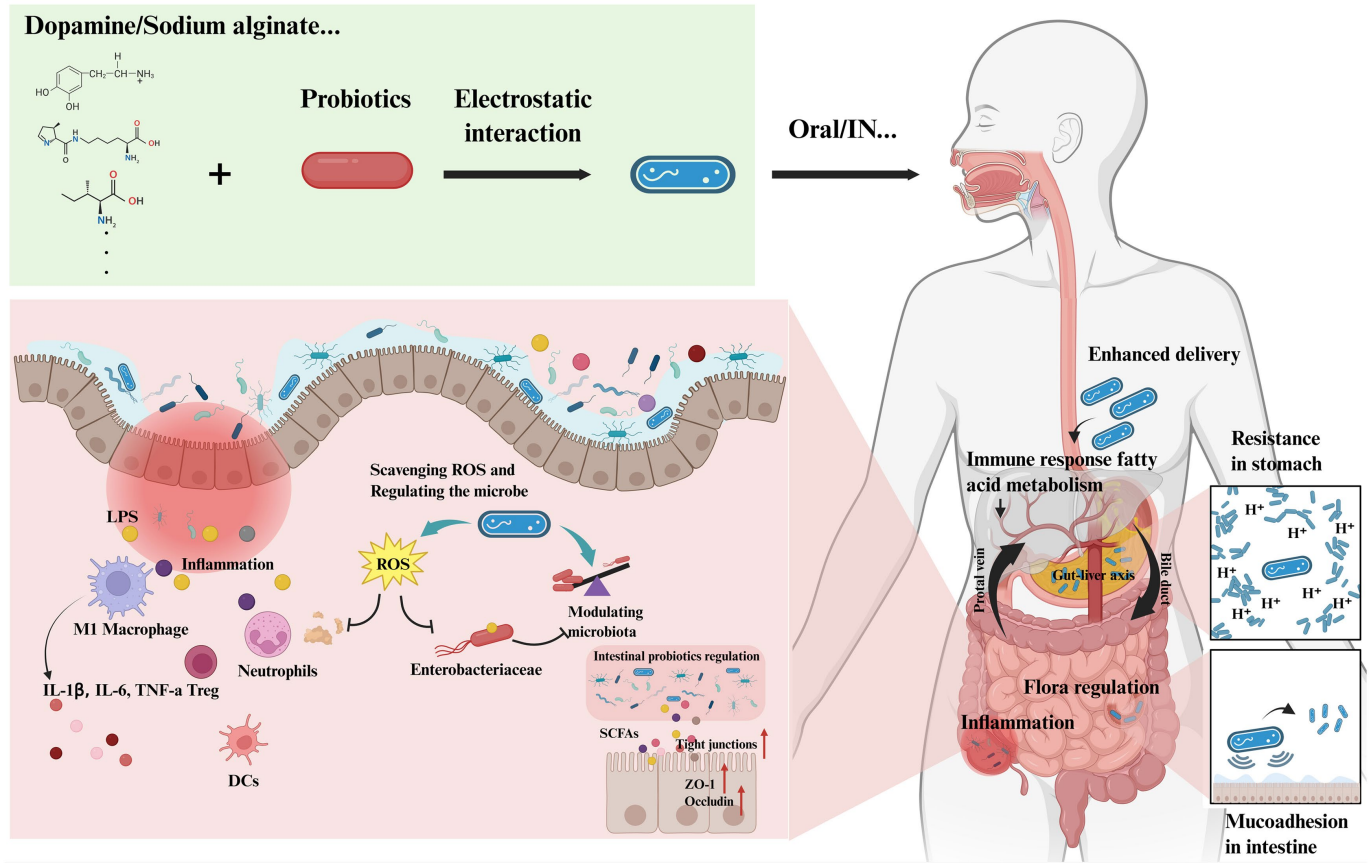
Surface modification technology significantly enhances the colonization capacity of probiotics. Probiotics are surface-modified with materials such as polydopamine, sodium alginate, and chitosan to increase their targeting specificity and adhesion capacity, thereby strengthening the ability of probiotics to alleviate or treat IBD. Created by Biorender.

Probiotic colonization efficiency depends on the ability of the bacteria to adhere to the intestinal mucosa. Surface modification techniques improve targeting and adhesion (Figure 6). Polydopamine combined with chitosan significantly increases probiotic colonization by promoting intestinal mucosa adhesion (Zhao et al., 2025). Probiotics modified with these materials show increased intestinal abundance and prolonged residence time, strengthening therapeutic effects (Pan et al., 2021).

Genetic modification also enhances targeting. Engineering probiotics to express specific adhesion molecules facilitates colon-specific colonization. Genetically modified strains expressing mucosa-binding adhesion proteins exhibit increased colon colonization, reducing loss in nontarget regions (Leeflang et al., 2025).

#### 4.2.2 Responsive delivery systems

**Figure 7:**
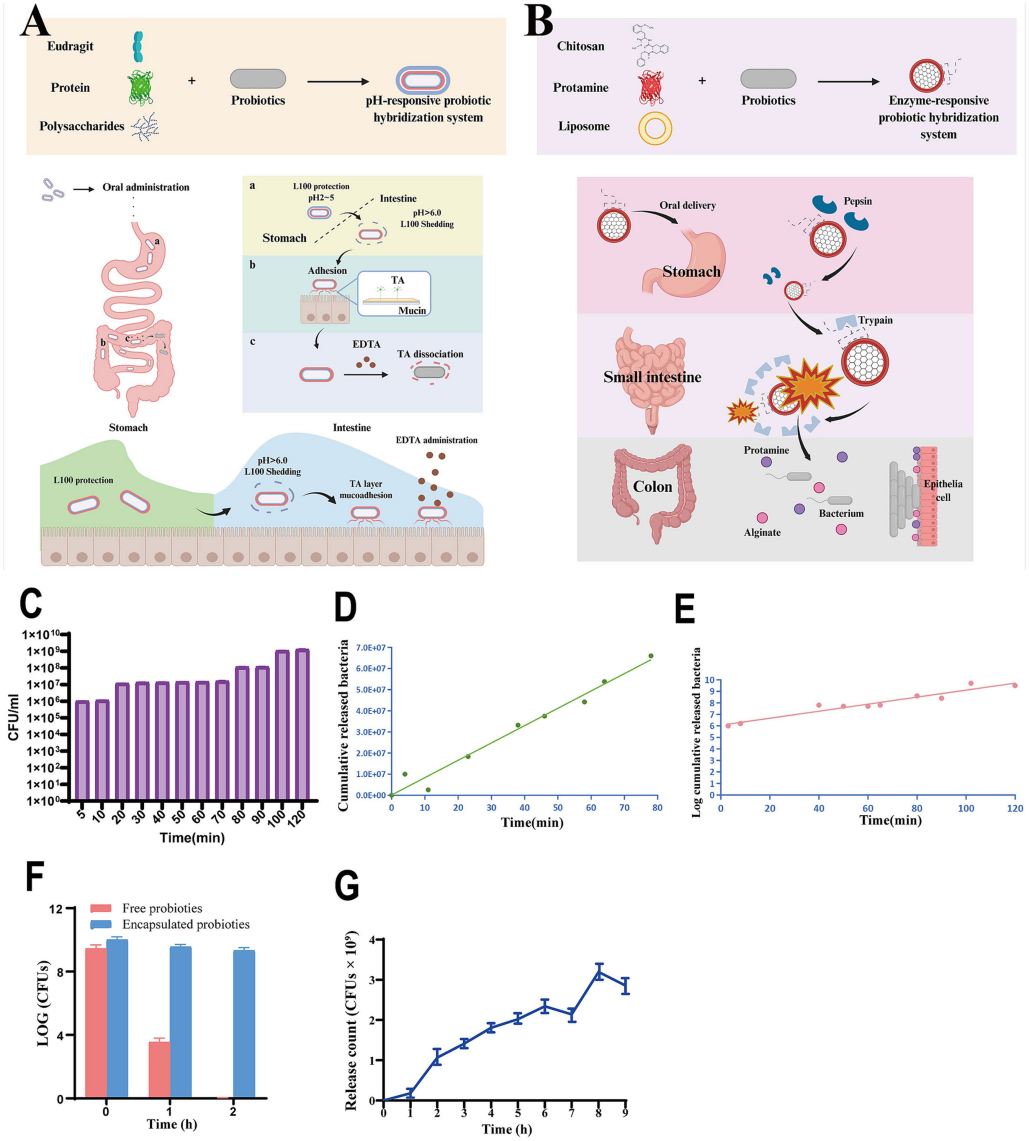
pH-responsive **(A)** and enzyme-responsive **(B)** delivery systems are important strategies for improving the colonization efficiency of probiotics. **(C)** The number of *EcN* released from microencapsulated *EcN* during 120 min incubation in Na-citrate buffer. The release kinetics models. **(D)** Zero-order kinetics model; **(E)** first-order kinetics model. **(F)** Viable *L. plantarum* counts when free *L. plantarum* and wood scroll encapsulated *L. plantarum* samples subjected to SGF for 2 h, revealing the protective capabilities of the wood scroll structure. **(G)** Cumulative amounts released when exposed to SIF for 9 h [data from Mawad et al. (2018) and Luan et al. (2022)]. Created by Biorender.

pH-responsive and enzyme-responsive delivery systems enable targeted probiotic release at specific gastrointestinal sites, improving colonization efficiency (Figure 7) (Dong et al., 2019; Hoang et al., 2021). pH-responsive microcapsules remain stable in acidic stomach/small intestine environments and degrade at neutral colonic pH, releasing probiotics precisely in the colon. Enzyme-responsive systems use polymer materials sensitive to gut enzymes to release probiotics in designated regions, leveraging localized enzymatic activity for precise delivery.

Novel delivery systems outperform encapsulation in targeting precision, enabling site-specific release in the colon or inflamed lesions—addressing encapsulation’s limitation of broad, nonspecific protection (Zhao et al., 2025). Compared with the effects of standalone encapsulation or unmodified probiotics, the synergistic effects of surface modification and responsive release yield higher colonization efficiency. Compared with genetic engineering, delivery systems avoid GMO-related safety and regulatory burdens, accelerating clinical translation. Their modular design supports integration with diverse probiotics and therapeutic agents, offering greater flexibility than strain-specific genome modification does. Furthermore, responsive systems adapt to dynamic gastrointestinal microenvironments (pH fluctuations, enzyme gradients)—a capability that genetic engineering can only replicate with complex, resource-intensive gene circuit design.

Unlike genetic engineering, delivery systems do not enhance the intrinsic functions of probiotics—they optimize delivery but not performance. Limited responsiveness to mild microenvironmental changes means that they often require robust signals to trigger release, potentially missing the signals necessary for release in subclinical lesions. Compared with encapsulation, delivery systems face more severe material–probiotic compatibility issues; some responsive polymers may exhibit cytotoxicity or disrupt probiotic metabolism. The complexity of manufacturing multilayered microcapsules or enzyme-sensitive nanoparticles also increases production costs and scalability challenges, which are less prominent in encapsulation or genetic engineering.

### 4.3 Engineered probiotics

#### 4.3.1 Gene editing and synthetic biology modifications

Advancements in gene editing (e.g., CRISPR) and synthetic biology have enabled precise probiotic genome optimization to enhance stress resistance (Ali et al., 2024). The GroESL molecular chaperone system promotes protein folding and increases cell survival under stress. Engineered *Lactobacillus paracasei* overexpressing GroESL shows enhanced stress resistance and broader environmental adaptability (Desmond et al., 2004). Other strategies (gene knockout/overexpression) modulate metabolic pathways to improve intestinal adaptability (Wang et al., 2024).

Recent advances in CRISPR-based strain engineering have transcended traditional Cas9-mediated double-strand breaks (DSBs), with precision tools such as base editors (BEs) and prime editors (PEs) revolutionizing probiotic modification (Ali et al., 2024; Raschmanová et al., 2026). Cytosine and adenine base editors enable single-nucleotide substitutions without inducing DSBs, drastically reducing off-target effects and genomic rearrangements—key limitations of early CRISPR systems (Kim et al., 2019). For example, compared to traditional CRISPR editing, BE-mediated modification of the glutamate decarboxylase (GAD) gene significantly increased *γ*-aminobutyric acid (GABA) production by *Lactobacillus plantarum* (Wang X. L. et al., 2025; Wang Z. Y. et al., 2025). Prime editors further increase this precision by enabling targeted insertions, deletions, and substitutions of up to 40 bp, facilitating seamless integration of therapeutic cassettes without homologous recombination templates (Anzalone et al., 2022; Doman et al., 2023). These tools expand the scope of CRISPR from stress resistance to the *de novo* synthesis of IBD-tailored bioactive molecules.

Synthetic biology involves advanced probiotic engineering through modular, orthogonal genetic circuits that enable programmable, tunable functions (Jiang et al., 2025; Kim et al., 2023). Modular design allows the assembly of sensing, signal transduction, and output modules from diverse microbial sources, minimizing crosstalk with endogenous probiotic pathways. For instance, a quorum-sensing (QS)-regulated circuit in *Bifidobacterium breve* induces IL-10 expression only when colonization density thresholds are met, ensuring therapeutic release in inflamed colons (Zhang and Wang, 2024). Synthetic oscillators and toggle switches further enable temporal control of therapeutic genes, aligning probiotic function with IBD progression (Kramer et al., 2005)—addressing the static nature of traditional probiotic therapies.

Smart biosensor probiotics, a product of combined synthetic biology and CRISPR engineering techniques, can detect complex, multisignal IBD-related cues (Charbonneau et al., 2020; Rottinghaus et al., 2020). Beyond single-signal sensing, modern designs integrate sensors for inflammatory mediators (TNF-*α* and IL-6), reactive oxygen/nitrogen species (ROS/RNS), and gut barrier markers (zonulin) (Mao and Chen, 2022). An engineered *E. coli* Nissle 1917 strain with a dual-sensor circuit releases anti-TNF nanobodies only in the presence of two inflammatory signals, minimizing off-target effects (Hua et al., 2025). Enhanced sensor sensitivity now enables the detection of subclinical inflammation, addressing an unmet need for early IBD intervention (Biela et al., 2015).

Probiotic integration—a rational combination of engineered strains with complementary functions—addresses the multifactorial pathogenesis of IBD (Wang et al., 2024; Cho et al., 2025). This strategy deploys strains tailored for distinct tasks: one sensing inflammation and releasing anti-inflammatory agents, another promoting intestinal epithelial cell (IEC) proliferation, and a third inhibiting pathogens (Bermúdez-Humarán et al., 2024). Compared with single-strain therapy, a combinatorial cocktail of CRISPR-edited *Lactobacillus rhamnosus* GG (anti-inflammatory), *Bifidobacterium adolescentis* (mucosal repair), and engineered *E. coli* Nissle 1917 (pathogen inhibition) reduced colon shortening and decreased proinflammatory cytokine levels more effectively in DSS-induced colitis model mice (Lin et al., 2024). Success relies on interstrain compatibility and coordinated function, which are addressed via synthetic biology tools such as QS-based intercellular communication modules.

#### 4.3.2 Smart targeting via genetic circuits

Genetic engineering transforms probiotics into “smart” microorganisms with precise targeting (Jiang et al., 2025). Programmed genetic circuits allow probiotics to sense environmental signals and respond accordingly. For example, engineered *E. coli* Nissle 1917 survives even when the L-lactate concentration exceeds 5 mM and releases payloads on demand (Isabella et al., 2018; Praveschotinunt et al., 2019). In IBD treatment, these strains detect inflammatory signals and release anti-inflammatory agents or antioxidants at lesion sites, alleviating inflammation (Charbonneau et al., 2020).

Genetic engineering offers unparalleled molecular precision and functional customization compared with encapsulation and delivery systems, enabling probiotics to perform IBD-specific tasks that are impossible with passive protection or targeted delivery alone (Jiang et al., 2025). Smart biosensor probiotics dynamically respond to gut microenvironmental cues, providing specificity that delivery systems cannot match. Probiotic integration enables synergistic multistrain therapy for complex IBD phenotypes, outperforming single-strain encapsulation and delivery systems. Additionally, genetic modifications enhance the intrinsic survival of probiotics, reducing their reliance on external protection or delivery vehicles.

Unlike encapsulation and delivery systems, genetic engineering has significant GMO safety risks, including horizontal gene transfer to gut commensals or unintended mutations—risks negligible in non-GMO strategies. Regulatory hurdles are far more stringent, with extensive preclinical/clinical data required to demonstrate safety, delaying translation compared with non-GMO approaches. The long-term stability of engineered functions in complex gut environments is another critical issue; engineered probiotics must maintain modified traits amid microbiota competition, nutrient limitations, and host immune responses—challenges that encapsulation and delivery systems mitigate via physical protection or controlled release. Furthermore, gene circuit design and genome modification require specialized expertise and resources, making genetic engineering less accessible than encapsulation or basic delivery systems for many research teams.

### 4.4 Biosafety considerations for engineered probiotics

The clinical translation of engineered probiotics is tightly constrained by biosafety concerns, as genetic modifications or foreign payloads may introduce unintended risks to the host, the gut microecosystem, or even the broader environment. These risks are amplified in IBD patients, who often have compromised intestinal barriers and dysregulated immune systems—making them more susceptible to microbial perturbations. Below is a detailed analysis of core biosafety risks, evidence-based mitigation strategies, and unresolved challenges that are supported by recent preclinical and early clinical data.

#### 4.4.1 Horizontal gene transfer (HGT): mechanisms, risks, and mitigation

HGT—the transfer of genetic material between nonparent-offspring microorganisms—is among the most critical biosafety risks associated with engineered probiotics. In the human gut, HGT occurs at frequencies 10–100 times greater than those in other environments because of three key factors: (1) high bacterial density (10^1^–10^12^ cells/g feces) facilitating cell-to-cell contact; (2) the presence of mobile genetic elements (MGEs, e.g., plasmids, transposons, and bacteriophages) that act as “vectors” for gene transfer; and (3) intestinal metabolites that transiently increase bacterial membrane permeability, promoting DNA uptake (Vos et al., 2024; Ali et al., 2024). With respect to engineered probiotics, HGT of foreign genes to pathogenic gut bacteria could lead to the development of “superbugs” with enhanced virulence or drug resistance, posing severe public health threats.

To address this risk, three evidence-based mitigation strategies have been validated in preclinical models, with varying degrees of translational potential.

##### 4.4.1.1 Chromosomal integration of engineered genes

Unlike plasmids (extrachromosomal DNA) that replicate independently and are easily transferred via conjugation, chromosomal integration embeds foreign genes into the probiotic’s genome, greatly reducing HGT risk. This is typically achieved via CRISPR-Cas9-mediated homologous directed repair (HDR), which targets specific genomic loci to insert engineered cassettes (Ali et al., 2024). A landmark study by Ali et al. (2024) compared HGT frequencies between plasmid-based and chromosomally integrated *Lactobacillus acidophilus* strains both expressing a green fluorescent protein (GFP) reporter. *In vitro* cocultures with a clinical isolate of *E. coli* (strain ATCC 25922) revealed that the HGT frequency of *L. acidophilus* was 1.2 × 10^−5^ events per cell, whereas the HGT frequency of chromosomally integrated *L. acidophilus* was <1 × 10^−7^ events per cell.

In a mouse model of DSS-induced colitis, fecal sampling over 4 weeks confirmed that no horizontal transfer of the chromosomal GFP cassette to native gut bacteria occurred. However, this strategy has several limitations; chromosomal integration can reduce the expression level of engineered genes and requires strain-specific HDR optimization.

##### 4.4.1.2 Suicide plasmids for transient gene expression

Suicide plasmids are engineered to replicate only under specific “permissive” conditions and degrade rapidly when these conditions are removed—ensuring that they are not retained or transferred long term. A recent example by Rahmati et al. (2025) involved a temperature-sensitive suicide plasmid (pTS-SOD) designed for *Lactococcus lactis*: this plasmid contains a replication origin (oriT) functional only at 30–37° C, whereas at temperatures >37.5° C, oriT is inactivated, and the plasmid is degraded via a built-in restriction enzyme (EcoRI) that cleaves the plasmid backbone.

*In vitro*, *L. lactis* carrying pTS-SOD retained the plasmid for 72 h at 30° CC but lost >99% of the plasmids within 24 h at 37° C. In a rat model of TNBS-induced colitis, fecal samples collected 7 days post-administration showed no detectable pTS-SOD (via PCR), confirming the “suicide” phenotype of the plasmid. This strategy is particularly useful for short-term therapeutic payloads but is unsuitable for long-term colonization due to plasmid loss.

##### 4.4.1.3 Auxotrophic markers instead of antibiotic resistance genes

In traditional plasmid-based engineering, antibiotic resistance genes are often used to select for transformed probiotics. However, these genes are high risk for HGT, as they can confer drug resistance to pathogens. Auxotrophic markers—genetic modifications that render the probiotic dependent on an exogenous nutrient for survival—eliminate this risk. The most widely used auxotrophic marker is thyA gene knockout (encoding thymidylate synthase), which prevents the probiotic from synthesizing thymidine (a critical nucleotide for DNA replication) (Isabella et al., 2018).

Isabella et al. (2018) engineered *E. coli* Nissle 1917 (a clinically validated probiotic) with thyA knockout and a chromosomally integrated IL-10 cassette. *In vitro*, the engineered strain (ECN-ΔthyA-IL10) only grew in media supplemented with 50 μg/mL thymidine; without thymidine, >99% of the cells died within 48 h. In a mouse model of colitis, ECN-ΔthyA-IL10 colonized the gut for 14 days when the mice received thymidine-supplemented drinking water (0.1 mg/mL) but was completely cleared within 7 days of thymidine withdrawal. Importantly, no HGT of the thyA knockout or IL-10 cassette was detected in native gut bacteria. Other auxotrophic markers have shown similar safety profiles but require strain-specific nutrient optimization.

#### 4.4.2 Containment strategies: ensuring spatial and temporal control

Containment strategies go beyond HGT mitigation to ensure that engineered probiotics do not persist in the gut or environment outside of therapeutic needs. These strategies are classified into two categories: passive containment (dependence on exogenous factors) and active containment (response to endogenous gut signals). Both aim to limit the probiotic’s lifespan to the duration of IBD activity, reducing long-term ecological impact.

##### 4.4.2.1 Passive containment: nutrient or temperature dependence

Nutrient-dependent containment is the most mature passive strategy, but temperature-dependent systems have gained traction for their ability to respond to host physiology (e.g., fever during IBD flares). Rahmati et al. (2025) developed a temperature-activated kill switch for *Lactococcus lactis* that combines two components. The first is a heat-inducible promoter (P_hsp_) derived from *Bacillus subtilis*, which is activated at temperatures >39° C (a common fever threshold in IBD flares), and the second is a cytotoxic gene (cidA) encoding a pore-forming protein that disrupts the bacterial cell membrane when expressed.

*In vitro*, *L. lactis* carrying the kill switch (LL-TKS) remained viable at 37° C (normal body temperature) but had a 99.9% mortality rate within 6 h at 39° C. In a mouse model of DSS-induced colitis (in which mice developed transient fever up to 39.5° C), LL-TKS was cleared from the gut within 48 h of fever onset, whereas a control strain (without the kill switch) persisted for 10 days. This strategy is advantageous for “on-demand” containment during flares but has several limitations: it does not activate in afebrile IBD patients and requires combination with nutrient-dependent systems for full coverage.

##### 4.4.2.2 Active containment: response to endogenous gut signals

Active containment systems use gut-specific signals to trigger probiotic elimination—aligning containment with disease activity. A ROS-responsive kill switch for *E. coli* Nissle—leveraging the high ROS levels in the inflamed IBD mucosa (10–50 μM H₂O₂, compared to <5 μM in the healthy gut)—uses a peroxide-inducible promoter (P_oxr_A) that is activated by H₂O₂, and when activated, P_oxr_A drives the expression of sulA, a gene that inhibits bacterial cell division (leading to apoptosis) (Charbonneau et al., 2020).

*In vitro*, *E. coli* Nissle with the ROS kill switch (ECN-ROS-KS) survived in low-ROS media (<5 μM H₂O₂) but stopped dividing and died within 12 h in high-ROS media (25 μM H₂O₂). In a DSS mouse model, ECN-ROS-KS colonized the inflamed colon for 7 days (during peak ROS production) and was cleared within 5 days as inflammation resolved (ROS levels decreased to <8 μM). This strategy is “self-regulating” but requires further validation in human IBD patients, where ROS levels vary between UC and CD (higher in UC colonic mucosa than in CD small bowel mucosa; Ashique et al., 2023).

#### 4.4.3 Ecological impact: balancing therapeutic efficacy and microbiome homeostasis

The gut microbiota is a complex ecosystem with overlapping metabolic and functional networks; introducing engineered probiotics could disrupt this balance via three mechanisms: (1) competition for nutrients or adhesion sites with native beneficial bacteria; (2) secretion of antimicrobial molecules (e.g., bacteriocins) that inhibit nontarget microbes; and (3) alteration of gut metabolite profiles. Assessing ecological impact requires long-term (≥12 weeks) studies using multiomics approaches (16S rRNA sequencing, metagenomics, and metabolomics) to capture both taxonomic and functional changes.

##### 4.4.3.1 Taxonomic impact: effects on beneficial and pathogenic taxa

Most preclinical studies have focused on short-term (4–8 weeks) ecological effects, with mixed results. The effect of *E. coli* Nissle expressing IL-10 (ECN-IL10) in DSS-treated mice was evaluated, and the results revealed no significant changes in the abundance of the beneficial taxa *Faecalibacterium prausnitzii* (0.8 ± 0.2% vs. 0.7 ± 0.1% in controls) or *Akkermansia muciniphila* (1.2 ± 0.3% vs. 1.1 ± 0.2% in controls), while the abundance of the pathogenic taxon *E. coli* (including AIEC) decreased 30%, likely due to nutrient competition. With respect to alpha diversity, the Shannon index (a measure of microbial diversity) remained unchanged in the context of ECN-IL10 treatment, with values of 4.2 ± 0.3 (ECN-IL10 group) versus 4.1 ± 0.2 (control group) (Praveschotinunt et al., 2019).

However, a longer-term study (16 weeks) by Sang et al. (2025) revealed that *Lactococcus lactis* expressing bacteriocins (LL-BAC) caused a 25% reduction in the abundance of *Bifidobacterium* in mice—likely because of the cross-reactivity of the bacteriocins with nontargeting gram-positive bacteria. This highlights the need for strain-specific antimicrobial payloads.

##### 4.4.3.2 Functional impact: metabolic and immune perturbations

Functional changes can be more impactful than taxonomic changes, as they directly affect host physiology. Analysis of the fecal metabolomes of mice treated with *Lactobacillus paracasei*-overexpressing GroESL (LP-GroESL) revealed that butyrate levels increased by 15% (from 2.1 ± 0.3 mM to 2.4 ± 0.2 mM), likely because of enhanced LP-GroESL survival and fermentation of dietary fiber; secondary bile acids decreased by 20%, which may reduce gut epithelial damage (a known effect of secondary bile acids in IBD); and lipopolysaccharide (LPS) levels decreased by 30%, which is consistent with reduced colonic inflammation (Wang et al., 2024).

In contrast, another study revealed that *E. coli* Nissle expressing SOD (ECN-SOD) caused a 10% reduction in acetate levels—possibly due to competition with native acetate-producing bacteria (Cao et al., 2023). This underscores the need for “metabolic profiling” of engineered probiotics to ensure that unintended metabolite shifts do not exacerbate IBD symptoms (Lynch et al., 2022).

##### 4.4.3.3 Unresolved challenges in ecological risk assessment

Ecological impact assessment still has two key gaps: interindividual variability and the absence of long-term human data. For instance, the composition of the gut microbiota varies widely among IBD patients, and an engineered probiotic that is safe in one patient may disrupt the composition of the microbiota in another. Additionally, most studies are in mice, and human data are limited to short-term (4–8 weeks) phase I trials (Praveschotinunt et al., 2019), which are insufficient to capture delayed effects. Future studies should use “microbiome rebound” assays—measuring how quickly the gut microbiota returns to baseline after the cessation of engineered probiotic treatment—to assess the reversibility of any perturbations.

## 5 Application of engineered probiotics in IBD treatment

### 5.1 Application of engineered probiotics in ulcerative colitis

#### 5.1.1 Performance of specifically genetically modified probiotic strains in preclinical models

UC is a chronic inflammatory condition that primarily affects the colonic mucosa and is characterized by persistent inflammation and ulceration of the intestinal mucosa. In recent years, genetically engineered probiotics have shown significant potential for UC treatment. Through genetic modification, *Lactococcus lactis* strains have been engineered to express anti-inflammatory cytokines, such as IL-10, which markedly alleviated colonic inflammation in animal models (Steidler et al., 2000). These engineered strains modulate the intestinal immune response by reducing the production of proinflammatory cytokines, thereby alleviating the symptoms of UC engineered. Studies have demonstrated that engineered probiotics constructed through synthetic biology techniques can sense inflammatory signals in the gut and release therapeutic enzymes, such as superoxide dismutase (Cao et al., 2023). In UC mouse models, this engineered probiotic significantly reduced oxidative stress levels and promoted intestinal mucosal healing. Further research has demonstrated that these engineered probiotics offer significant advantages in reducing inflammation and promoting tissue repair.

#### 5.1.2 Progress and challenges in clinical trials

Despite the promising results obtained in preclinical studies, the clinical application of engineered probiotics in the treatment of UC still faces several challenges. At present, some therapeutic strategies based on engineered probiotics have progressed to clinical trials. In particular, preliminary clinical safety evaluations have been completed for a *Lactococcus lactis* strain engineered to express IL-10, and the results demonstrated good tolerability (Braat et al., 2006). However, the therapeutic efficacy of this strain requires further validation in larger trials. Key challenges encountered in clinical trials include ensuring the long-term stability of engineered strains, improving their ability to colonize the human gut, and avoiding adverse immune responses. Moreover, maintaining therapeutic function within the complex intestinal environment while avoiding irreversible effects on the host microbiome remains a critical issue to be addressed (Sang et al., 2025).

### 5.2 Applications of engineered probiotics in Crohn’s disease

#### 5.2.1 Targeted strategies for inflamed sites in Crohn’s disease

Crohn’s disease (CD) is a chronic inflammatory disorder affecting the entire intestinal wall. Targeted therapy at inflammatory sites has become a research hotspot in therapeutic development (An et al., 2014; Centanni et al., 2024). Using genetic engineering techniques, probiotics can be modified to express specific adhesion molecules, enabling selective adherence to the inflamed intestinal regions of patients with CD. For instance, a genetically modified Bifidobacterium strain has been engineered to express adhesins that recognize inflammation-associated glycoproteins, demonstrating strong targeting capabilities in a CD mouse model. Another promising strategy involves engineering probiotics to produce enzymes capable of degrading proinflammatory mediators (Barra et al., 2020). For example, an engineered probiotic that has been constructed to secrete proteases can specifically degrade cytokines implicated in the inflammatory cascade, thereby alleviating intestinal inflammation. This targeted approach not only enhances therapeutic efficacy but also minimizes the side effects of drugs in noninflamed regions.

#### 5.2.2 Applications of engineered probiotics in combination therapy schemes

In the treatment of CD, engineered probiotics are commonly used in combination with other therapeutic modalities to improve overall efficacy. Specifically, engineered probiotics can be combined with immunosuppressants or biologic agents (Torres et al., 2020; Wu et al., 2024). This combination therapy can more effectively regulate intestinal immune responses and promote the repair of the intestinal mucosa. Moreover, combining engineered probiotics with nutritional interventions such as the administration of short-chain fatty acids can improve the gut microenvironment, enhance probiotic colonization and improve therapeutic outcomes (Lynch et al., 2022; Wang X. L. et al., 2025; Wang Z. Y. et al., 2025). This multipronged therapeutic strategy offers new insights for the personalized treatment of CD.

## 6 Personalized intervention strategies

### 6.1 Probiotic customization on the basis of individual gut microbiota characteristics

#### 6.1.1 Application of gut microbiota detection techniques in personalized interventions

The development of gut microbiota detection techniques, combined with machine learning-driven multiomics integration frameworks, provides powerful and essential tools for personalized interventions. High-throughput sequencing enables detailed analyses of the composition and functional activity of individual gut microbiota (Gibbons et al., 2022; Valdés-Mas et al., 2024), while multiomics data (including gut microbiome, host genomics, transcriptomics, metabolomics, and proteomics) are integrated via machine learning algorithms to decode complex host–microbe interactions (Li M. F. et al., 2024; Li M. M. et al., 2024; Zhang et al., 2025). These integrated frameworks not only identify dominant and low-abundance microbial populations with their metabolic products but also quantify the microbial–metabolic–host signaling networks that underpin IBD pathogenesis. Currently, 16S rRNA gene sequencing and metagenomic sequencing, coupled with machine learning-based multiomics integration, are widely utilized to generate high-resolution gut microbiota profiles. This approach further enables the identification of IBD-specific microbial signatures and their functional correlates, laying a robust scientific foundation for refining personalized intervention plans.

#### 6.1.2 Selection of appropriate engineered probiotics on the basis of individual microbiota characteristics

Precision medicine, which involves the development of personalized treatment plans based on individual genetic features, environmental factors, and lifestyles, is being increasingly anchored in machine learning-driven multiomics integration frameworks for IBD treatment (Ceccato et al., 2025; Mao and Chen, 2022). These frameworks integrate gut microbiota multiomics data with host genomic and phenotypic information to construct predictive models that predict patient responses to specific engineered probiotics. Machine learning algorithms can be used to analyze high-dimensional multiomics datasets and identify nonlinear correlations between microbial/host signatures and treatment outcomes (Mao and Chen, 2022; Wu and Gadsden, 2023). This enables the prioritization of probiotic formulations most likely to modulate host–microbe interactions favorably, thereby improving clinical responses. Additionally, precision medicine leverages real-time multiomics monitoring combined with machine learning to dynamically track therapeutic responses, allowing timely adjustments to probiotic formulations for optimal efficacy (Wei et al., 2025).

### 6.2 The potential of precision medicine in IBD treatment

#### 6.2.1 Concept of precision medicine and its alignment with IBD treatment

Precision medicine is a medical model that involves the development of personalized treatment plans on the basis of individual genetic features and environmental and lifestyle factors (Ceccato et al., 2025). In IBD treatment, precision medicine is primarily reflected in the formulation of personalized probiotic intervention strategies based on the patient’s gut microbiota characteristics and disease phenotypes (Mao and Chen, 2022). By integrating gut microbiota data with genetic profiles, it is possible to predict patient responses to specific probiotics. These predictive models aid in selecting the engineered probiotics most likely to be effective, thereby improving clinical outcomes. Additionally, precision medicine involves monitoring the patient’s therapeutic responses, enabling timely adjustments to ensure optimal efficacy.

#### 6.2.2 Development and implementation of personalized probiotic intervention strategies

The development of personalized probiotic intervention strategies requires comprehensive consideration of the patient’s gut microbiota characteristics, disease phenotype, and therapeutic responses (Goldiș et al., 2025). On the basis of patient gut microbiota data, engineered probiotics simultaneously expressing anti-inflammatory cytokines and antimicrobial peptides can be developed (Ma et al., 2022). These probiotics exhibit dual functions: reducing inflammation and inhibiting the growth of harmful bacteria, thereby improving the patient’s gut microenvironment. During the implementation of personalized probiotic intervention strategies, close monitoring of therapeutic response is crucial. Regular fecal microbiota analysis can evaluate changes in the gut microbiota composition, probiotic colonization and effectiveness (Barra et al., 2020; Wu et al., 2025). Moreover, monitoring clinical symptoms and biomarkers is necessary for timely treatment adjustments. This personalized intervention strategy offers new insights and methods for the treatment of IBD.

### 6.3 Challenges and opportunities for personalized probiotic interventions

#### 6.3.1 Technological innovations driving the development of personalized interventions

Technological innovation is a key factor driving the development of personalized probiotic interventions. Continuous progress in gene editing, synthetic biology, and high-throughput sequencing technologies has allowed researchers to design and optimize engineered probiotics with greater precision (Dong et al., 2019). CRISPR-Cas9 gene-editing technology, in particular, enables precise modification of the probiotic genome, facilitating the production of a variety of therapeutic molecules (Rahmati et al., 2025). Moreover, synthetic biology techniques can be employed to construct sophisticated gene circuits, enabling probiotics to sense environmental signals and respond appropriately (Cao et al., 2023). These technological innovations provide powerful tools for personalized probiotic interventions, allowing them to better meet the needs of individual patients.

#### 6.3.2 Challenges and future research directions

Although personalized probiotic interventions have shown great potential in the treatment of IBD, their clinical application still faces several challenges. Accurately predicting patient responses to probiotic treatments remains a primary difficulty. Although several predictive models have been developed, their predictive ability and reliability still require further validation (Dong et al., 2019; Arosa et al., 2024). Additionally, ensuring that engineered probiotics retain their therapeutic functionality within the complex intestinal environment without causing irreversible disturbances to the host microbiota remains a critical challenge. Future research should focus on developing more precise predictive models, optimizing the design and function of engineered probiotics, and exploring new therapeutic targets and mechanisms. Comprehensive studies addressing the long-term safety and stability of engineered probiotics are necessary to establish a scientific foundation for the widespread clinical application of personalized probiotic interventions.

## 7 Regulatory landscape and future directions

The clinical translation of engineered probiotics for IBD is shaped by a complex interplay of regulatory frameworks, manufacturing feasibility, and scientific priorities. As a class of novel biologics with genetic modifications or synthetic components, engineered probiotics face stricter oversight than conventional probiotics do—reflecting concerns about long-term safety, ecological impact, and consistent therapeutic performance. Below is a detailed analysis of the current regulatory landscape, manufacturing and economic challenges, and actionable future research directions to accelerate their clinical adoption.

### 7.1 Regulatory standards for engineered probiotics

Engineered probiotics are universally classified as biologics by regulatory agencies, but their classification subcategories and approval requirements vary by region. These differences arise from varying risk assessments of genetic modification, with a focus on three core pillars: (1) preclinical safety profiling; (2) clinical evidence of efficacy and safety; and (3) manufacturing quality and consistency.

#### 7.1.1 United states food and drug administration (FDA)

The FDA regulates engineered probiotics under the Public Health Service Act (PHS Act) and Federal Food, Drug, and Cosmetic Act (FD&C Act), with two primary pathways depending on the degree of genetic modification. For nongenetically modified engineered probiotics, the FDA typically requires an Investigational New Drug (IND) application for clinical trials, followed by a New Drug Application (NDA) if approved. For genetically modified (GM) probiotics, the FDA mandates a Biologics License application (BLA)—the highest standard for biologics—due to their novel molecular composition (Asai et al., 2022).

Key requirements for the BLA of GM probiotics include preclinical safety data; clinical trial design; and chemical, manufacturing, and control (CMC) data. Among these, preclinical safety data include the risk of HGT, acute, subchronic, and chronic toxicity, immunogenicity, and genetic stability, and these data require comprehensive evaluation. Clinical trials include phase I trials, phase II trials, and phase III trials. Phase I trials (*n* = 20–100 healthy volunteers or mild IBD patients) are conducted to evaluate safety and pharmacokinetics (e.g., gut colonization duration via qPCR); phase II trials (*n* = 100–300 IBD patients) are conducted to evaluate dose–response relationships and preliminary efficacy (e.g., Mayo score reduction for ulcerative colitis [UC]); and phase III trials (*n* = 300–1,000 IBD patients) are conducted to confirm long-term efficacy (12–24 months) and rare adverse events. CMC data include strain construction, fermentation process validation, purification and formulation, and quality control methods. These data require the submission of detailed documents.

Notably, the FDA’s 2023 Guidance for Industry: Probiotics for Human Use (FDA-2023-D-0945) emphasizes “risk-based oversight”—GM probiotics with high HGT potential require additional containment data, whereas low-risk strains may qualify for expedited pathways such as breakthrough therapy designation (BTD) if they address unmet needs.

#### 7.1.2 European medicines agency (EMA)

The EMA classifies GM-engineered probiotics as advanced therapy medicinal products (ATMPs)—specifically, gene therapy products—under regulation (EC) No 1394/2007. This classification reflects their “novel mode of action” (genetic modification to deliver therapeutic effects) and triggers a specialized approval process managed by the Committee for Advanced Therapies (CAT) in collaboration with the Committee for Medicinal Products for Human Use (CHMP).

Key ATMP requirements distinct from those of the FDA include ecological impact assessment, assessment of ATMPs, and long-term clinical follow-up (LTCF). An ecological impact assessment is mandatory for assessing the potential environmental persistence of genetically modified (GM) probiotics, as well as their ability to transfer genes to environmental bacteria.

An ecological impact assessment is needed to evaluate the potential persistence of genetically modified (GM) probiotics in the environment and their capacity for gene transfer to environmental bacteria. The EMA’s 2022 guidelines on environmental risk assessment for ATMPs (EMA/CHMP/ATMP/483044/2016) require modeling of environmental concentrations and “worst-case scenario” testing. Postapproval requires LTCF to be conducted, aiming to track delayed adverse events and ecological impacts. The EMA mandates a risk management plan (RMP) with predefined endpoints. Prior to IND submission, sponsors must seek scientific advice from the CAT to align study design with ATMP standards. Formal classification confirmation is also required to confirm ATMP status, avoiding postsubmission delays.

For non-GM-engineered probiotics, the EMA follows the traditional centralized procedure (valid in all EU/EEA countries) with requirements similar to those of the FDA but with greater emphasis on “patient-centered outcomes”.

#### 7.1.3 National medical product administration (NMPA)

The NMPA of China regulates engineered probiotics under the regulations of the administration of medical products (2019) and aligns with international standards while imposing the following unique requirements for local relevance: (1) manufacturing facilities must meet cleanroom grade A/B standards for GM strain handling, with negative-pressure labs to prevent cross-contamination; the NMPA’s 2024 technical guidelines for the evaluation of gene therapy products mandate real-time monitoring of fermentation and QC testing of every batch; (2) phase II/III trials must include ≥50% Chinese IBD patients to account for ethnic differences in gut microbiota composition and drug metabolism; multicenter trials must be conducted at NMPA-accredited sites; and (3) the NMPA requires a 3-year post-marketing surveillance (PMS) program with active pharmacovigilance and periodic reevaluation of manufacturing processes.

### 7.2 Manufacturing and economic considerations

The scalability and cost of engineered probiotics are critical bottlenecks for clinical translation, as their complex production processes are far more resource intensive than those of conventional probiotics. Below is a detailed breakdown of the challenges and mitigation strategies.

#### 7.2.1 Scalability challenges

Scalability is defined as the ability to produce consistent, high-quality batches at volumes sufficient for clinical use (10,000–100,000 doses/year) while maintaining viability and genetic stability.

##### 7.2.1.1 Fermentation of GM probiotics

Compared with wild-type strains, GM strains often exhibit reduced fitness and require optimized nutrient media and strict temperature/pH control to prevent plasmid loss or off-target mutations. Large-scale bioreactors (500–5,000 L) exacerbate these challenges, as gradients in oxygen concentration or nutrient distribution can lead to batch-to-batch variability. GM probiotics require biosafety level 2 (BSL-2) fermentation facilities to prevent environmental release, including dedicated air filtration systems (HEPA filters) and wastewater decontamination. Compared with conventional probiotic facilities, these requirements increase capital expenditure (CAPEX).

##### 7.2.1.2 Targeted encapsulation technologies

Laboratory-scale encapsulation (1–10 g batches) uses electrostatic droplet generators or microfluidics to produce uniform particles, but scaling to 1–10 kg batches requires parallelization of the equipment and optimized drying processes. For example, spray drying—common for conventional probiotics—reduces the viability of GM probiotics because of heat stress, necessitating low-temperature lyophilization, which increases energy consumption. The polydopamine-chitosan coating of probiotics requires precise control of the pH and reaction time to avoid aggregation. Scaling this process to 100 L batches requires stirred-tank reactors with inline pH monitoring, increasing process complexity and QC costs.

##### 7.2.1.3 Downstream processing

The purification of GM probiotics requires the removal of fermentation byproducts and the confirmation of GM allele purity. This involves ultrafiltration and flow cytometry sorting, which reduce yield by 20–30% and add 1–2 days to the production time compared with conventional centrifugation-based purification methods.

#### 7.2.2 Cost-reduction strategies

##### 7.2.2.1 Process optimization

Continuous fermentation: Replacing batch fermentation with continuous processes reduces batch-to-batch variability by 50% and increases productivity. Replacing expensive PLGA with natural polysaccharide blends maintains 80–90% encapsulation efficiency while reducing material costs by 80%.

##### 7.2.2.2 Regulatory and policy levers

Seeking FDA BTD or EMA PRIME designation reduces R&D time by 1–2 years and cutting costs by 20–30%. For example, a GM probiotic for refractory UC could qualify for BTD if it demonstrates a 50% remission rate in phase II. Collaborations between academic labs, biotechnology companies, and governments can share R&D costs.

## 8 Conclusion and outlook

This review outlines the current state and recent progress in the use of engineered probiotics for treating IBD. Through genetic modification and synthetic biology, these probiotics produce various therapeutic molecules, regulating the balance of the gut microbiota, strengthening intestinal barrier function, and modulating immune responses (Steidler et al., 2000; Zhou et al., 2021). Notably, their development and clinical translation are increasingly empowered by ongoing advances in systems biology, multiomics technologies (genomics, transcriptomics, metabolomics), and artificial intelligence (AI), which enable more precise identification of patient-specific microbial and immune dysfunctions—an essential prerequisite for targeted IBD management. Engineered probiotics exhibit significant therapeutic potential for UC and CD, with three key advantages: targeted delivery to inflamed areas (lowering side effects in noninflamed regions; Sang et al., 2025), synergistic application with other therapies to potentially enhance efficacy, and the capacity for personalized interventions. Specifically, the integration of multiomics data and AI-driven analysis may decipher individual microbial–immune dysregulation patterns, thereby optimizing treatment outcomes and reducing adverse effects, although this requires further clinical validation (Abeltino et al., 2024; Li M. F. et al., 2024; Li M. M. et al., 2024).

Despite their potential, the following critical challenges remain: improving the survival and colonization of engineered probiotics in the complex intestinal microenvironment; ensuring their long-term safety and stability without inducing irreversible perturbations to the host microbiota; and validating the accuracy, reliability, and generalizability of AI-powered predictive models for patient therapy responses. While systems biology and multiomics technologies offer promising approaches to address these issues, their practical implementation remains challenging. Thus, optimizing probiotic formulations to align with individual patient profiles, guided by systems biology and AI-driven multiomics analysis, remains a key future research direction (Zhang et al., 2025; Yadav and Shukla, 2020).

Advancements in gene editing, synthetic biology (for building complex genetic circuits), and high-throughput sequencing have propelled engineered probiotics into a new phase of development. Personalized strategies may further expand their application scope, offering a potentially safer and more tailored alternative to traditional therapies that are often associated with significant side effects. While targeted and personalized approaches involving engineered probiotics are unlikely to completely transform IBD treatment paradigms in the near term, they may emerge as important components of future IBD care. Their successful translation could help reduce unnecessary medical resource use and advance precision/personalized IBD treatment toward a potential clinical gold standard—provided that the current technical and safety challenges are adequately resolved.
